# Dynamic silencing of somatic L1 retrotransposon insertions reflects the developmental and cellular contexts of their genomic integration

**DOI:** 10.1186/s13100-017-0091-2

**Published:** 2017-05-10

**Authors:** Manoj Kannan, Jingfeng Li, Sarah E. Fritz, Kathryn E. Husarek, Jonathan C. Sanford, Teresa L. Sullivan, Pawan Kumar Tiwary, Wenfeng An, Jef D. Boeke, David E. Symer

**Affiliations:** 1 Department of Biological Sciences, Birla Institute of Technology and Science Pilani, Pilani, 333031 Rajasthan India; 2 Laboratory of Immunobiology, Mouse Cancer Genetics Program and Basic Research Laboratory, Center for Cancer Research, National Cancer Institute, Frederick, MD 21702 USA; 3 Present Address: Birla Institute of Technology and Science, Pilani, Dubai campus, Dubai, United Arab Emirates; 4 Department of Cancer Biology and Genetics, The Ohio State University, Columbus, OH USA; 5 Department of Internal Medicine, The Ohio State University, Columbus, OH USA; 6 Biomedical Sciences Graduate Program, The Ohio State University, Columbus, OH USA; 7 Present Address: National Heart, Lung and Blood Institute, National Institutes of Health, Bethesda, MD USA; 8 Present Address: Aventiv Research, Inc., Columbus, OH USA; 9 Present Address: Drug Safety Research and Development, Pfizer, Inc., Groton, CT USA; 10 Present Address: Biocon, Bangalore, India; 11 Department of Molecular Biology and Genetics, The Johns Hopkins University School of Medicine, Baltimore, MD USA; 12 Present Address: Department of Pharmaceutical Sciences, South Dakota State University, Brookings, SD USA; 13 Present Address: Institute for Systems Genetics, New York University Langone Medical Center, New York, NY USA; 14 Human Cancer Genetics Program, and Department of Biomedical Informatics, The Ohio State University, Columbus, OH USA; 15 Human Cancer Genetics Program, Department of Cancer Biology and Genetics, and Department of Biomedical Informatics, The Ohio State University, Tzagournis Research Facility, Room 440, 420 West 12th Ave, Columbus, OH 43210 USA

## Abstract

**Background:**

The ongoing mobilization of mammalian transposable elements (TEs) contributes to natural genetic variation. To survey the epigenetic control and expression of reporter genes inserted by L1 retrotransposition in diverse cellular and genomic contexts, we engineered highly sensitive, real-time L1 retrotransposon reporter constructs.

**Results:**

Here we describe different patterns of expression and epigenetic controls of newly inserted sequences retrotransposed by L1 in various somatic cells and tissues including cultured human cancer cells, mouse embryonic stem cells, and tissues of pseudofounder transgenic mice and their progeny. In cancer cell lines, the newly inserted sequences typically underwent rapid transcriptional gene silencing, but they lacked cytosine methylation even after many cell divisions. L1 reporter expression was reversible and oscillated frequently. Silenced or variegated reporter expression was strongly and uniformly reactivated by treatment with inhibitors of histone deacetylation, revealing the mechanism for their silencing. By contrast, *de novo* integrants retrotransposed by L1 in pluripotent mouse embryonic stem (ES) cells underwent rapid silencing by dense cytosine methylation. Similarly, *de novo* cytosine methylation also was identified at new integrants when studied in several distinct somatic tissues of adult founder mice. Pre-existing L1 elements in cultured human cancer cells were stably silenced by dense cytosine methylation, whereas their transcription modestly increased when cytosine methylation was experimentally reduced in cells lacking DNA methyltransferases DNMT1 and DNMT3b. As a control, reporter genes mobilized by *piggyBac* (*PB*), a DNA transposon, revealed relatively stable and robust expression without apparent silencing in both cultured cancer cells and ES cells.

**Conclusions:**

We hypothesize that the *de novo* methylation marks at newly inserted sequences retrotransposed by L1 in early pre-implantation development are maintained or re-established in adult somatic tissues. By contrast, histone deacetylation reversibly silences L1 reporter insertions that had mobilized at later timepoints in somatic development and differentiation, e.g., in cancer cell lines. We conclude that the cellular contexts of L1 retrotransposition can determine expression or silencing of newly integrated sequences. We propose a model whereby reporter expression from somatic TE insertions reflects the timing, molecular mechanism, epigenetic controls and the genomic, cellular and developmental contexts of their integration.

**Electronic supplementary material:**

The online version of this article (doi:10.1186/s13100-017-0091-2) contains supplementary material, which is available to authorized users.

## Background

Approximately half of the human and mouse genomes is comprised of various classes of transposable elements (TEs). These TE insertions have mobilized by distinct mechanisms and accumulated over evolutionary time [1–4]. Until recently, such mobilization was thought to occur almost exclusively in germline cells or early in embryogenesis [5]. However, recent studies established that L1 retrotransposons, along with other classes of mobile genetic elements, also can move actively in somatic cells, i.e., in mouse, rat and human neural progenitor cells, in the developing brain, and in certain human cancers [6–11].

This ongoing movement of endogenous TEs including L1 retrotransposons can result in diverse genetic consequences. These include insertional and deletional (indel) gains and losses of genomic fragments, exon shuffling, insertional mutagenesis of genes, probably chromosomal translocations and inversions, and expression of retrotransposon-initiated fusion transcripts (RIFTs), among others [12–22]. Much of our existing knowledge about TE-related genetic disruption was derived from specific examples of *de novo* insertions causing diseases in mouse and man [23–25]. By contrast, the epigenetic marks established at newly mobilized TEs have not been well characterized.

Cytosine methylation is a key epigenetic regulatory mark localized predominantly within extant L1 retrotransposons and other TEs in mammalian genomes. It has been strongly associated with their transcriptional silencing and regulation, and may affect expression of adjacent genes [26, 27]. Cytosine methylation can be inherited either through mitotic or meiotic cell divisions, and in general are stably maintained. In normal somatic cells, L1 retrotransposons are heavily methylated at CpG dinucleotides, but in most cancers they become hypomethylated, potentially resulting in increased transcription and mobilization [9, 28–30].

A recent study of host epigenetic responses to L1 retrotransposition in various somatic cells including embryonal carcinoma (EC) cells showed that newly integrated L1 reporters were silenced by *de novo* transcriptional gene silencing (TGS) [31]. The epigenetic modifications at newly inserted L1 retrotransposons included histone deacetylation, but not *de novo* cytosine methylation. By contrast, more strongly repressive epigenetic marks including cytosine methylation have been identified at recently inserted L1 elements that were transmitted via meiotic cell division through the mouse germ line in a transgenic mouse model [32]. Similarly, reporter genes that were transduced by retrovirus mobilization or integrated randomly as a transgene typically were methylated rapidly after integration in mammalian cells [33, 34]. Such silencing has been associated with the source and sequence content of the reporter genes themselves.

In classic examples of variable epigenetic silencing at mammalian TEs, changes in epigenetic marks (e.g., methylcytosine density) at pre-existing, integrated endogenous retroviruses (ERVs) have resulted in highly variable expression of nearby genes, resulting in variable phenotypes in genetically identical siblings. Variable but heritable phenotypes in the classic pseudo-agouti mouse model illustrate this impact of epigenetic regulation of an existing TE on neighboring gene expression [35–37]. The term “variegation” describes this epigenetically mediated variability in phenotypes. Typically such phenotypic variation is due to the relatively unstable inheritance of epigenetic controls at a so-called “metastable epiallele”, from a cell to its daughter cells [38]. A related and profoundly important question asks how widespread and functionally significant are the heritable impacts of TEs on gene expression and regulation [39].

To investigate how different cellular contexts and mechanisms of transposition may impact reporter expression and epigenetic silencing of newly mobilized TE insertions, we developed new and highly sensitive real-time reporters. We compared the reporter expression and silencing of newly integrated L1 insertions vs. of new *piggyBac* (*PB*) insertions. We corroborated recently reported results about cytosine methylation established at new L1 integrants transmitted through the mouse germ line [32]. We observed variable reporter expression and epigenetic controls established at newly integrated L1 elements that mobilized in different genomic, cellular and developmental contexts.

## Results

### A sensitive, real-time reporter for L1 retrotransposition reveals dynamic silencing of new genomic insertions

To define the genetic consequences of *de novo* L1 retrotransposition, several research groups have engineered L1 donor constructs to track mobilization [19, 40–42]. In each of these retrotransposition assays, the L1 donor was marked in its 3′ untranslated region (UTR) by a reporter gene disrupted by an artificial intron (AI). Identification of the spliced reporter gene in host genomic DNA indicated that the newly integrated element had undergone expression and splicing as an RNA intermediate, confirming that it was a bona fide product of L1 retrotransposition [40].

To assess expression of L1 reporters that are newly mobilized by retrotransposition, we constructed novel, real-time reporter assays. Their expression levels would not be influenced by positive or negative selective pressures imposed on the cells. We first chose *TEM1*, encoding a beta-lactamase, to generate an exquisitely sensitive reporter assay in living cells. Its expression levels can be quantified over a very large dynamic range extending over four orders of magnitude (Additional file 1: Figure S1: [43]). This greatly exceeds sensitivity of other real-time reporters used in L1 retrotransposition assays. We also chose green fluorescent protein (GFP) [41] as a second, convenient but less sensitive reporter for particular assays. Mimicking the design of other retrotransposition reporter constructs, we introduced the AI into donor cassettes to disrupt the *TEM1* or GFP open reading frames (ORFs), respectively. The AI would be spliced from L1 RNA transcripts at the time of retrotransposition, so a newly integrated reporter cDNA would lack the AI and therefore could be expressed as the intact gene [40].

**Fig. 1:**
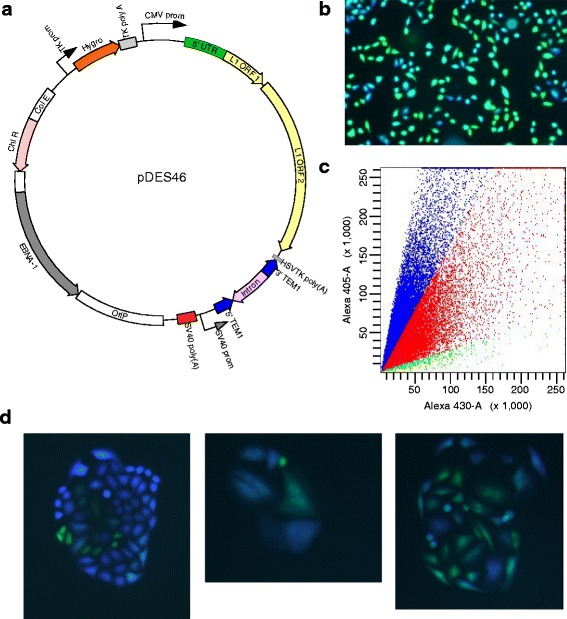
A sensitive real-time reporter reveals variable and dynamic silencing of L1 integrants in cultured cells. **a** Schematic of a human L1 retrotransposon donor plasmid, pDES46. L1.3 was tagged at its 3′ end with a highly sensitive reporter gene, beta-lactamase (*TEM1; blue open read frames*) [19], interrupted by an artificial intron (AI; *pink*). This L1 donor construct, based on the pCEP4 episomal plasmid, was stably maintained on Hygromycin selection. Upon L1 mobilization, expression of real-time beta-lactamase reporter (encoded by the spliced, integrated *TEM1* gene) was screened (without selection). **b** Fluorescence microscopy reveals wide-ranging levels of beta-lactamase (TEM1p) expression, ranging from zero or low (*green cells*) to high (*blue*) levels. HeLa cells were transfected with pDES46. Later the bulk population of cells was stained using a fluorescent substrate for the beta-lactamase reporter, CCF2-AM. **c** Scatter plot from flow cytometry, performed on a subclone of cells harboring L1 reporter integrants. Fluorescence emissions were detected for (*y-axis, 405 nm emission*) blue and (*x-axis, 430 nm*) green individual CCF2-AM-stained cells, as well all intermediate expression levels (*red*). **d** Variegation of L1 reporter expression in individual cell subclones. HeLa cells were transfected with pDES46, subcloned by limiting dilution so that all cells in a colony would contain the same *de novo* L1 reporter integrants, and stained with CCF2-AM and visualized by fluorescence microscopy

We marked the full-length, retrotransposition competent, human L1.3 retrotransposon with a novel *TEM1*-AI reporter cassette. The resulting construct was subcloned into a stably maintained, episomal vector, i.e., pCEP4, resulting in pDES46 (Fig. 1a). Cultured human cancer cells, i.e., HCT116 and HeLa, were transfected. To assure stable maintenance of the donor plasmid episome, transfected cells were selected for Hygromycin resistance. In parallel we introduced a transient plasmid expressing GFP alone, to measure transfection efficiency.

To measure beta-lactamase expression levels as a surrogate for active retrotransposition, we incubated transfected cells with CCF2-AM fluorescent substrate. Two enzymatic activities are required in this real-time assay. First, constitutive cellular esterases cleave the –AM esters, resulting in trapping of the charged CCF substrate in the cytoplasm (Additional file 1: Figure S1A). Next, the reporter beta-lactamase cleave the beta-lactam ring in CCF, disrupting fluorescence resonance energy transfer (FRET) and changing the fluorescence emission wavelength from green to blue. After incubation with CCF2-AM, cells were examined by fluorescence microscopy [43]. Uncleaved CCF2 inside the cells would fluoresce green, implying low or absent beta-lactamase expression. In turn, this indicated either no retrotransposition, integration of only truncated TEM1 reporter gene, or silencing of the newly integrated reporter gene (Additional file 1: Figure S1). The presence of blue fluorescence indicated high levels of beta-lactamase expression and therefore robust L1 retrotransposition without silencing of the newly integrated reporter gene (Fig. 1b; Additional file 1: Figure S1).

We confirmed that L1 retrotransposition was the mechanism of reporter mobilization and integration in the transfected cells. We used PCR to amplify the integrated *TEM1* reporter gene across the spliced (excised) AI junction [13, 40], using primers DES657 and DES658 (Additional file 1: Table S1). To determine the detailed structures of several diverse integrants, we employed a PCR-based assay (Additional file 1: Figure S2 and Table S2) [44]. First, we restricted genomic DNA using common 4 bp-cutting restriction endonucleases. Appropriate adaptors were ligated onto compatible overhangs, and PCR primers annealing to the L1 and the adaptor respectively were used [22, 44]. Insertion-host genomic junctions were recovered as PCR products and were assessed by Sanger sequencing, resulting in the recovery of 9 independent integrant sites. Although several integrants were not long enough to include any L1 sequence or even full-length reporter genes per se, they nevertheless were *bona fide* L1 retrotransposition events (Additional file 1: Figure S2 and Additional file 1: Table S2), as their structures included target site duplications (TSDs), a poly(A) tail, and occasional 5′ inversions [13, 45, 46]. At an L1 integrant on chromosome 2, an intact, spliced *TEM1* gene had been inserted (Additional file 1: Figure S2). Its spliced structure and its TSDs confirmed that it had been retrotransposed.

After selection on Hygromycin for 10 d, bulk populations of transfected cells were screened for beta-lactamase expression by treating them with CCF2-AM (Fig. 1b). Fluorescence microscopy revealed that the beta-lactamase expression was “variegated”. Some individual cells fluoresced bright blue, indicating robust expression of beta-lactamase as the reporter for the occurrence of at least one retrotransposition event. Other immediately adjacent cells in the same colony appeared green, indicating that the integrated reporter gene was not expressed and therefore was potentially silenced. Many other cells displayed various intermediate shades of blue and green, implying partial silencing or less-than-maximal expression of the *TEM1* reporter (Fig. 1b).

To quantify *TEM1* reporter expression in individual cells incubated with CCF2-AM, we used flow cytometry [47]. Wide-ranging fluorescence emissions ranging from green to blue were visualized using fluorescence microscopy for individual cells in the bulk population of transfected cells (Figs. 1b and c), and quantified using flow cytometry. Blue/green ratios were calculated as a surrogate score for beta-lactamase activity [43]. These ratios ranged from <10 to well over 150, thereby demonstrating a large dynamic range over which beta-lactamase was differentially expressed in individual cells (Fig. 1c; Additional file 1: Figure S1). To standardize the blue and green signals in flow cytometry, all-green and all-blue cell populations also were assayed in each experiment (Additional file 1: Figure S3).

To test heritability of reporter expression or silencing through multiple rounds of mitotic cell division, we isolated individual cells from these mixed populations by limiting dilution, and then grew up subcloned progeny cells. The resulting cellular clones were stained with CCF2-AM and then visualized by fluorescence microscopy (Fig. 1d). Resulting subcloned daughter cells recurrently stained various shades of blue and green, documenting continued variability in reporter expression. Occasional colonies contained mostly blue cells, indicating high levels of reporter expression amongst most of the cloned daughter cells. However, even in such predominantly blue subclones, occasional green cells arose, indicating the stochastic and dynamic establishment of reporter silencing. Upon a second round of cell subcloning by sequential limiting dilution, again we observed mitotically heritable patterns of reporter expression, revealing mostly stable (blue) or variable (mixed) expression of L1 reporter integrants. This result again suggested that while the states of reporter expression or silencing were mostly heritable, they also could oscillate (Fig. 1d). This variability in expression implied that the newly inserted L1 reporters are epigenetically regulated.

**Fig. 2:**
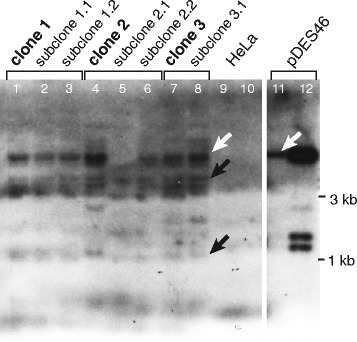
Southern blot of subcloned cell lines expressing various levels of beta-lactamase reporter reveals their genetic similarity. HeLa cells were transfected with pDES46. To evaluate reporter expression, they were stained with CCF2-AM and examined by fluorescence microscopy. Individual, subcloned cells were derived by limiting dilution. For Southern blots, DNA was extracted, 10 mcg was restricted with EcoRI, electrophoresed on a gel, blotted, and probed for *TEM1* reporter. *Reporter expression phenotypes:* lanes 1–6, clones 1 and 2 and their derived subclones, variegating phenotype with mixed beta-lactamase (*TEM1*) expression; lanes 7–8, clone 3 and its derived subclone, predominantly blue cells with high levels of beta-lactamase (*TEM1*) expression. *Cell line names*: Clone 1: cell line 5B 0.3c/w C9 (parent of sub-clones corresponding to lanes 2 and 3)‬‬‬‬‬‬; subclone 1.1: 5B #1G10; subclone 1.2: ‬‬‬‬‬‬5B #4D9‬‬‬‬‬‬; clone 2: 6I 0.3c/w C8 (parent of sub-clones corresponding to lanes 5 and 6)‬‬‬‬‬‬; subclone 2.1: 6I #8G2‬‬‬‬‬‬; subclone 2.2: 6I #9 F8‬‬‬‬‬‬; clone 3: 1 1c/w B11 (parent of sub-clone in lane 8)‬‬‬‬‬‬; subclone 3.1: 1 #5H5‬‬‬‬‬‬. *Control samples*: lanes 9–10, negative, untransfected HeLa cells; and lanes 11–12, pDES46 plasmid DNA (50 and 500 pg). *White arrows*: *TEM1* bands shared between cell clones and pDES46 L1 donor plasmid; *black arrows*, integrated *TEM1* bands present in all clones, but not in EcoRI- digested pDES46 ‬‬‬‬‬‬‬‬‬‬‬‬‬‬‬‬‬‬‬‬‬‬‬‬‬‬‬‬‬‬‬‬‬‬‬‬‬‬‬‬‬‬‬‬‬‬‬‬‬‬‬‬‬‬‬‬‬‬‬‬‬‬‬‬‬‬‬‬‬‬‬‬‬‬‬‬‬‬‬‬‬‬‬‬‬‬‬‬‬‬‬‬‬‬‬‬‬‬‬‬‬‬‬‬‬‬‬‬‬‬‬‬‬‬‬‬‬‬‬‬‬‬‬‬‬‬‬‬‬‬‬‬‬‬‬‬‬‬‬‬‬‬‬‬‬‬‬‬‬‬‬‬‬‬‬‬‬‬‬‬‬‬‬‬‬‬‬‬‬‬‬‬‬‬‬‬‬‬‬‬‬‬‬‬‬‬‬‬‬‬‬‬‬‬‬‬‬‬‬‬‬‬‬‬‬‬‬‬‬‬‬‬‬‬‬‬‬‬‬‬‬‬‬‬‬‬‬‬‬‬‬‬

To assess whether the variable L1 reporter expression could be attributed to variable numbers and locations of newly integrated L1 reporters, we used Southern blotting to investigate eight related cell lines that had been subcloned from the same initial population of HeLa transfectants (Fig. 2). These clones and their subclones displayed different levels of L1 reporter expression, i.e., either mostly high (staining blue), or variegated (mixed, oscillating) cells including many that were green (i.e., low beta-lactamase expressing). A radiolabeled probe specific for the β-lactamase reporter was used to detect the reporter gene copies in the clones. Almost all subclones shared 7 or more bands of various molecular weights, including a dominant one similar in size to the donor pDES46 episome. Occasional single bands also were detected in a few of the individual clones (Fig. 2), without apparent correlation with expression levels, suggesting gains or losses of additional retrotransposition events. Although the reporter expression levels diverged markedly between these cell clones, the pattern of *TEM1* bands was mostly similar amongst the cell clones and subclones. This suggested that the variable reporter expression was likely due to epigenetic variation between the clones, since their genetic variation appeared to be limited.

### Lack of de novo cytosine methylation at new L1 integrants in cultured cancer cells

In previous studies of human L1 retrotransposition in cultured cancer cells, expression of the integrated *Neo* ^*R*^ reporter was enforced by positive selection [13, 40]. Selection with the drug G418 imposed a requirement for strong expression of the *Neo* resistance gene, since cells lacking it would be killed. Thus we reasoned that epigenetic marks observed at newly retrotransposed *Neo* ^*R*^ reporters could have been in favor of active, euchromatic marks.

**Fig. 3:**
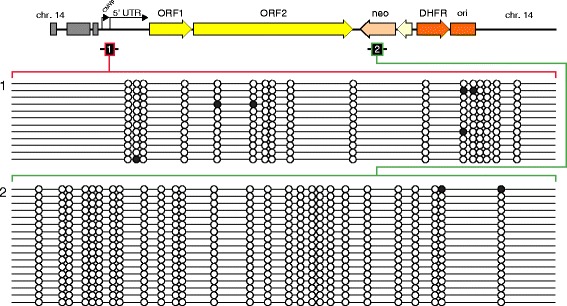
Lack of cytosine methylation at full-length L1 insertions in cultured cancer cells. *Top*: Schematic of a full-length *de novo* human L1.3 insertion (in chr. 14 in derivative cell clone 7H2 after transfection of the HCT116 parental cell line, as previously mapped [13]). *Black bars*: PCR amplicons analyzed by bisulfite sequencing. Minimal *de novo* cytosine methylation was observed at the newly retrotransposed (1) 5' transduced sequence from the distal CMV promoter and proximal L1 5' UTR; and (2) in the spliced *Neo* reporter gene. PCR amplicons were generated using primers DES512 x DES530 (1); and DES515 x DES524 (2), respectively. Similar results were obtained at a second independent full-length L1 integrant on chromosome 3 in the 2A2 subclone of transfected HCT116 cells [13]

In addition to finding many truncated *de novo* L1 insertions, we previously mapped two full-length L1 insertions, on chrs. 2 and 14 of transfected HCT116 cells [13]. These newly inserted sequences that were retrotransposed by L1 each included 5′ transduction of adjacent 5′ CMV promoter fragments. Their identification provided us with a unique opportunity to study *de novo* cytosine methylation, established both at the inserted reporter and several kilobases upstream in the 5′ transduced sequences and in the proximal L1 5′ UTR. We measured DNA methylation using conventional bisulfite conversion followed by PCR amplification and sequencing. We found virtually no DNA methylation at the 5′ UTR of both insertions, as only 2.9 and 0.4% of all CpG dinucleotides at those locations were methylated, respectively (Fig. 3). In addition, as expected, the spliced reporter integrants at the 3′ ends of these full-length L1 integrants also were almost entirely unmethylated; only 0.4% of all their CpG dinucleotides were methylated (Fig. 3).

To confirm that the host cells still harbored effective maintenance methyltransferase activity, we measured cytosine methylation within the proximal portion of 5′ UTRs of pre-existing genomic L1Hs elements. They were densely methylated (~64% on average, Additional file 1: Figure S4), confirming that methylated CpG dinucleotides (meCpG) are maintained in the cultured cancer cells. The bisulfite sequencing assay may underestimate unmethylated cytosine content at CpG dinucleotides, as many cytosines undergo spontaneous deamination over time [48, 49], resulting in TpG dinucleotides. Such pre-existing mutations are indistinguishable from unmethylated CpG dinucleotides upon treatment with bisulfite.

### Impact of genome-wide hypomethylation on expression of endogenous L1 elements

In mutant double knockout (DKO) HCT116 cells, which lack both the maintenance DNA methyltransferase gene *DNMT1* and the *de novo* methyltransferase *DNMT3b* [50], cytosine methylation at pre-existing L1 elements was markedly reduced. Only ~6.5% of all CpG dinucleotides remained methylated in DKO cells (Additional file 1: Figure S4), reflecting a ~90% reduction in L1 methylation.

Because cytosine methylation is a strong and stable mediator of transcriptional gene silencing (TGS) [27], we compared L1 transcript levels in DKO cells vs. their parental (wildtype) controls. We conducted expression profiling by performing long-read serial analysis of gene expression (LongSAGE) [51]. We corroborated the results using several other transcript profiling methods (manuscript in preparation). Transcription of pre-existing genomic L1 templates (measured at their 3′ ends) was only minimally detectable in HCT116 cells (Additional file 1: Table S3), as expected [52]. By contrast, L1 transcript levels were increased modestly (approximately 3-fold) in the DKO cells (Additional file 1: Table S3), consistent with derepression of the TGS regulating their expression.

### DNA methylation does not silence newly integrated L1 reporters in cultured cancer cells

We conducted bisulfite sequencing to examine cytosine methylation levels at several independently retrotransposed, spliced *TEM1* reporter integrants. After their retrotransposition, inserted L1 reporter sequences were retained in the host cell genomes in the absence of imposed positive selection. As was the case with L1-*Neo* ^*R*^ integrants (Fig. 3), both the integrated L1 reporter *TEM1* (including 18 CpG dinucleotides in an amplicon spanning the splice site in an artificial intron), and an L1 integrant including a portion of the SV40 promoter and *TEM1* reporter (including 20 CpGs) were almost completely unmethylated (Additional file 1: Figure S5). Therefore de novo cytosine methylation played no role in silencing or variegated expression of the TEM1 reporter in these cultured cancer cells (Fig. 1). These results corroborated what we observed after positive selection on *Neo* ^*R*^ (also driven by the SV40 promoter as described in [40] and [13]), suggesting that regardless of imposed selection, only minimal methylation is established at new L1 integrants.

Several of the newly integrated sequences retrotransposed by L1 that were recovered from HeLa cells had inserted into repetitive elements pre-existing in the host genome (Additional file 1: Table S2). Nevertheless, bisulfite sequencing analysis of new reporter insertions in bulk showed that most were unmethylated (data not shown). This result suggests that the epigenetic controls established at *de novo* L1 insertions do not reflect spreading of the repressive marks already maintained at neighboring, extant repetitive elements.

### Histone deacetylation is strongly associated with L1 reporter silencing in cultured cancer cells

**Fig. 4:**
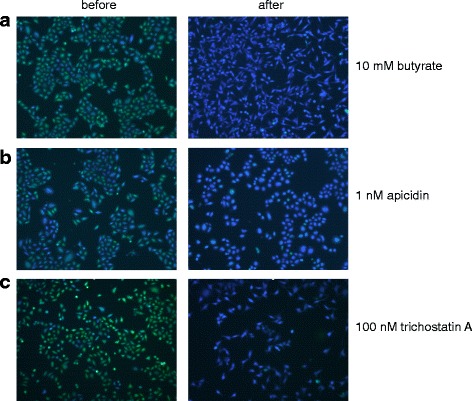
Variable L1 reporter expression in cultured cancer cells is associated with changes in histone acetylation. Cultured human cervical cancer **(**HeLa) cells harboring *de novo* L1 reporter integrants were assayed for reporter beta-lactamase expression by incubating them with the fluorescent substrate, CCF2-AM. *Left*: Before and *right*: after incubation for 24 h with various histone deacetylase inhibitors including: **a** 10 mM butyrate; **b** 1 nM apicidin; and **c** 100 nM TsA. Similar responses also were observed upon treatment for 24 h with 1 uM scriptaid and with 5 mM nicotinamide respectively (Additional file 1: Figure S6)

The heritability and the variability in L1 reporter expression in cultured cancer cells suggested that new L1 insertions are epigenetically silenced. To evaluate the possibility that histone tail lysine acetylation could be involved in L1 reporter silencing, we investigated several subcloned cell lines harboring reporter integrants whose expression was variegated or mostly repressed. We treated the cells with various histone deacetylase (HDAC) inhibitors including 100 nM trichostatin A (TsA), 10 mM sodium butyrate, 1 μM scriptaid, 1 nM apicin, and 5 mM nicotinamide (Fig. 4 and Additional file 1: Figure S6). Each of these agents was added in standard growth medium to the cultured cells. Upon incubation for 24 h, expression of the silenced reporter gene was reactivated in virtually all cells. Treated cells showed consistently high levels of *TEM1* reporter expression, as demonstrated by their uniform blue fluorescence upon staining with CCF2-AM (Fig. 4 and Additional file 1: Figure S6). Thus a broad range of HDAC inhibitors from different mechanistic categories was active in de-repressing the silenced reporters.

Upon withdrawal of the HDAC inhibitor TsA from derepressed cells by washout of the drug, silencing of the *TEM1* reporter was gradually re-established over 1 – 3 days (Additional file 1: Figure S7). This resetting of L1 reporter silencing demonstrated that it can be dynamically re-established and is reversible. In addition, the state of reporter expression generally appears to be heritable (Fig. 1d). Thus we conclude that the establishment and maintenance of L1 retrotransposon silencing in cultured human cancer cells is consistent with a *de novo* epigenetic mechanism involving dynamic changes in histone lysine deacetylation, but not cytosine methylation.

### New L1 integrants undergo rapid and dense DNA methylation in mouse ES cells

To study epigenetic controls at *de novo* L1 integrants in other cellular and developmental contexts, we induced new mobilization of a highly active synthetic L1 retrotransposon in mouse ES cells. The Bruce4 parental ES cell line [53] was transfected with linearized pJH435, resulting in an inducible, codon-optimized synthetic mouse L1 (smL1, ORFeus) donor present in the genome of the resulting Truck_305 cells [54]. We activated L1 retrotransposition by infecting the Truck_305 cells with an adenovirus expressing Cre recombinase, to remove the floxed beta-geo gene from their donor construct. This resulted in the juxtaposition of ORFeus ORF1 and ORF2 directly downstream of the CAG promoter, thereby activating smL! transcription and potentially their mobilization [54].

**Fig. 5:**
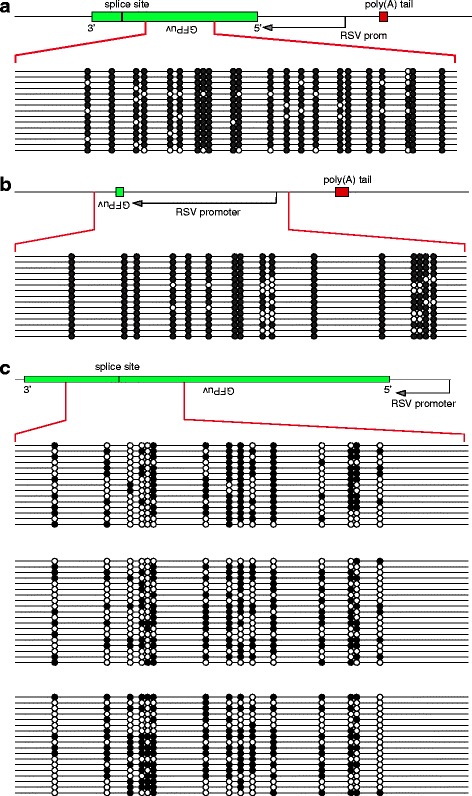
New L1 integrants in mouse embryonic stem (mES) cells undergo dense cytosine methylation. Dense cytosine methylation at new L1 integrants in mouse ES cells was revealed by bisulfite sequencing. Initially, Bruce 4 cells were transfected with pJH435, encoding an inactivated L1 ORFeus donor element marked with GFPuv-AI reporter regulated by a respiratory syncytial virus (RSV) promoter. Upon activation of L1 donor expression by transient infection of the culture using adenoviral Cre recombinase, individual colonies were picked and cultured on feeder cells for > 2 months. Of these mES subclones, most harbored newly retrotransposed L1 reporter integrants, as shown by a PCR-based assay documenting spliced integrated copies of the reporter GFPuv-AI (not shown). Their cytosine methylation status was assessed either in bulk or at individual loci using bisulfite sequencing. **a** For mES subclone 1B6-A07, we used primers DES3301 x DES3314, which anneal within the gene encoding GFPuv. This PCR amplicon does not cross the AI splice site, so unspliced donor L1 sequences also can be amplified. **b** For mES subclone 1B6-A08, primers DES3298 x DES3299 were used. **c** For mES clones 1B06/B02, 1C6 and 2D4, primers DES3321 x DES3322 were used to assay 15 CpG dinucleotides in a 234 nt amplicon across the GFPuv-AI splice junction

After exposing the Truck_305 mES cells to Cre, individual colonies were picked without regard to GFPuv expression, to derive subclonal populations potentially harboring new smL1 insertions. Genomic DNA was isolated from several of these mES cell clones. We did not routinely screen for GFPuv expression using excitation light in the UV range, because a high fluorescence background was observed in cultured cells. In addition, the UV light required to excite GFPuv would be expected to damage the cells when examined, so they could not be cultured further. Instead, linear amplification mediated-PCR (LAM-PCR) was performed to recover any new L1 ORFeus integrants, regardless of their expression of GFPuv. They were sequenced and mapped, and custom bisulfite sequencing primers were designed. Genomic DNA was modified with sodium bisulfite, and then PCR amplification was performed using primers internal to the reporter, or alternatively to target individual integrants. Amplicons were cloned and sequenced. The results showed heavy methylation of the retrotransposed reporter gene, assayed either in bulk or from individual L1 integrants (Fig. 5).

To evaluate silencing of a second, independent reporter gene, we also transfected pJL5, a donor plasmid encoding L1 ORFeus marked by *TEM1*-AI in its 3′ UTR, into mES cells (not shown). Minimal expression of integrated L1 reporters was observed, consistent with dense *de novo* cytosine methylation resulting in strong silencing. An alternative explanation for low beta-lactamase expression was that L1 ORFeus did not retrotranspose efficiently in mES cells. However, this same donor element mobilized very actively in various cultured cancer cell lines [19, 55]. Upon limiting dilution, we observed rare mES subclones that exhibited stable, robust reporter expression, suggesting derepressed newly retrotransposed reporter insertions. However, their DNA methylation was not investigated further.

### New L1 integrants undergo rapid, dense DNA methylation in various tissues in vivo

To study the epigenetic modifications established at new L1 integrants in vivo, we obtained several tissues from a transgenic mouse model, in which L1 ORFeus had retrotransposed initially in “pseudofounder” animals. Pseudofounder mice were defined as those mice in the first generation that harbored new, spliced L1 insertions but lacked the unspliced donor element. Their progeny also harbored some of the same, newly retrotransposed L1 ORFeus insertions as were present in the initial pseudofounders themselves, showing that these genomic L1 insertions could be transmitted through the germ line. The new L1 insertions likely had retrotransposed from the donor episome, immediately after its injection as a transgene and before its loss due to cell division during early embryogenesis [55].

**Fig. 6:**
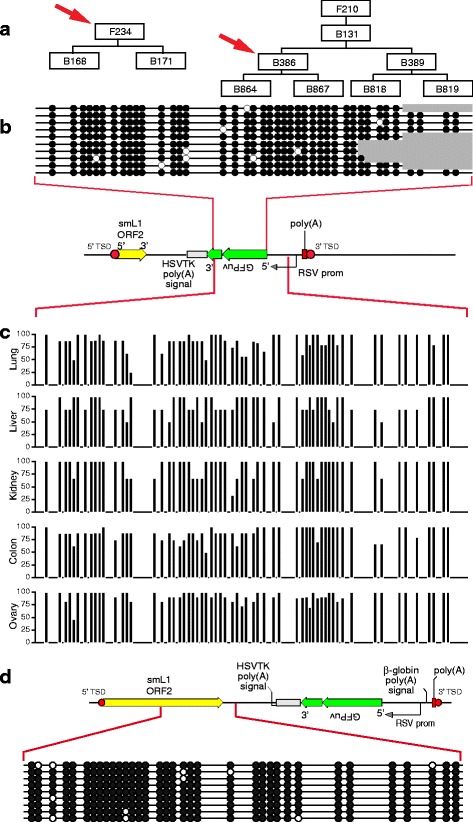
*De novo* silencing of somatic L1 insertions by dense cytosine methylation during embryogenesis. We evaluated cytosine methylation at new L1 insertions genome-wide by performing bisulfite sequencing. **a** Pedigrees of (*left*) pseudofounder mice F235 and F234, and (*right*) offspring mouse B386 (*red arrows*). At least some of the *de novo* L1 integrants initially integrated in pseudofounder mice, despite the absence of the L1 donor, were transmitted to their offspring. **b** Dense *de novo* methylation at a new L1 insertion in pseudofounder mouse F235, revealed by bisulfite sequencing using primers DES2219 x DES2221 (Additional file 1: Table S1). Each row represents an individual sequence read. *Filled circles*, methylcytosines; *open circles,* unmethylated cytosines. *Gray shading, right*: sequence data not available. *Bottom*: Schematic of *de novo* L1 integrant. Cytosine methylation within the spliced GFPuv reporter gene was assayed (*red connecting lines*). Other integrants at independent genomic loci also may have been assayed by the same amplicons. **c** Dense de novo methylation in various somatic tissues (*left*) in pseudofounder mouse F234, including lung, liver, kidney, colon and ovary, using the PCR amplicon DES2219 x DES2221. Data are presented as cumulative percentages methylated (*y-axis*) for 61 CpG dinuceotides at the indicated positions in genomic template DNA (*x-axis*). **d** Dense cytosine methylation at a new L1 insertion (*schematic,* top), initially identified in founder mouse F210, which was transmitted through the germ line to its offspring. Tail tissue from progeny mouse B386 (*panel A, right, red arrow*) was assayed using primers DES2016 x DES2018

We measured *de novo* cytosine methylation established at the newly mobilized L1 ORFeus integrants in the pseudofounders, as well as at some of the same integrants transmitted to offspring [55, 56]. Genomic DNAs isolated from various somatic tissues from members of three pedigrees were treated with sodium bisulfite and sequenced (Fig. 6). The results showed that almost all of the CpGs in independent *de novo* L1 integrants were methylated in two independent pseudofounder mice, F235 and F234 (Fig. 6), in a variety of somatic tissues including the tail and various internal organs.

We also used locus-specific primers to conduct bisulfite sequencing PCR at particular genomic targets (Fig. 6). Tail DNA samples from N2 generation mouse B386 and its progeny B864 and B867 (N3 generation) were used for this more focused study. As was the case with the bulk assays, almost all CpG dinucleotides at the specific genomic target sites were methylated (Fig. 6d). Dense cytosine methylation also was observed in other somatic tissues and at independent L1 insertions in mouse B389 and its offspring, as well as in other mice (Fig. 6; data not shown).

Taken together, these results demonstrated that: a) new L1 insertions occurring early in embryogenesis underwent dense, *de novo* methylation during development; b) methylation was maintained through differentiation into a range of tissues in the developing organism; and c) methylation at such new L1 insertions was maintained and/or re-established upon their transmission through the germline.

### PiggyBac reporters are not variegated or silenced in cultured cancer cells and mouse ES cells

**Fig. 7:**
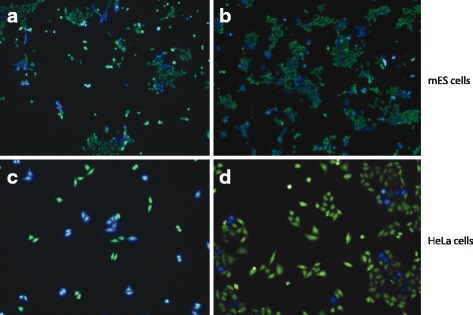
Lack of variegation and silencing of reporters newly mobilized by *PB* transposons. **a**, **b** Stable expression of *TEM1* beta-lactamase reporter mobilized by *PB* DNA transposon in mouse ES (mES) cells. E14Tg2a.4 cells were transfected with *PB* vectors carrying the TEM-1 reporter as cargo. Upon staining with CCF2-AM substrate, resulting mES cells fluoresced either blue (stable expression of reporter) or green (no reporter), with minimal variegation observed. **c**, **d** Stable expression of *TEM1* beta-lactamase reporter mobilized by *PB* in HeLa cells. No variegation was observed. Treatment with HDAC inhibitors did not increase the percentage of cells fluorescing blue (not shown), indicating a lack of silencing of reporter insertions that had been mobilized by *PB*

To compare reporter integrants mobilized by different mechanisms into distinct genomic targets, the same reporter genes, including *TEM1* and green fluorescent protein (GFP), were engineered to be mobilized as cargo by *PB* DNA transposons. A large majority of HeLa cells harboring newly transposed integrants displayed stable, robust expression of the reporters when mobilized by *PB* (Fig. 7). We observed a bimodal distribution of reporter expression, i.e., with individual cells displaying either robust expression or no expression (Fig. 7) and minimal or no variegation.

As a negative control, we transfected the reporter gene plasmid alone, without *PB* transposase. In resulting transfected cells, no integration events were detected, the transient donor plasmid harboring the unintegrated reporter gene was gradually lost, and no reporter gene expression was observed after several days in culture. In cells that had been transfected with *PB* transposase and the reporter donor, and then subcloned by limiting dilution, we occasionally observed a small fraction of the cells expressing stably diminished levels of the reporter protein.

In additional control experiments, we also launched *PB* transposons carrying comparable reporter genes in mES cells. As was observed in cancer cell lines, expression levels of integrated *PB* reporters in mES cells remained at high levels even after many days of culture (Fig. 7). No variegation or decreases in reporter expression were detected. Therefore, they underwent no or only minimal epigenetic silencing. Flow cytometry experiments confirmed a biomodal distribution of reporter expression in mES cells with *PB* transposition, indicating a lack of variegation and silencing of *PB* insertions (not shown), distinct from that observed after L1 retrotransposition.

## Discussion

Endogenous retrotransposons comprise a substantial portion of the mouse and human genomes. Several distinct TE families have modified the mammalian genome profoundly over evolutionary time [57, 58]. The genetic and genomic changes caused by endogenous mobilization of human or mouse L1 retrotransposons have been well studied. By contrast, the epigenetic regulation of *de novo* L1 integrants has not been evaluated fully in the wide-ranging biological contexts in which retrotransposition can occur [39, 59].

Here we investigated the expression and epigenetic silencing of newly integrated L1 reporters in cultured human cancer cells, mouse ES cells, and in several tissues of pseudofounder transgenic mice and their progeny. The results revealed distinctive patterns of L1 reporter expression and associated epigenetic marks, which are associated with the genomic, cellular and developmental contexts of mobilization.

Newly retrotransposed and integrated L1 reporters in cultured human cancer cells frequently were silenced. This silencing was heritable, as daughter cells tended to display levels of reporter expression that were similar to their parents after mitotic cell divisions (Fig. 1). However, L1 reporter expression also was variegated and oscillated dynamically, as it occasionally ranged from almost completely silenced to high levels of expression over just a single or a few cell divisions (Fig. 1). Despite virtually identical patterns of L1 insertoins at a genetic level, cell clones displayed marked differences in phenotypes, suggesting epigenetic regulation of reporter expression (Fig. 2). New L1 reporter integrants remained almost completely unmethylated, even after many cell divisions, regardless of their expression levels (Fig. 3). L1 reporters were silenced rapidly by histone tail deacetylation, as shown by strong, uniform reactivation of reporter expression upon treatment with any of several diverse HDAC inhibitors (Fig. 4, Additional file 1: Figure S6). Histone deacetylation-mediated L1 silencing was re-established within 2–3 days in most cells upon removal of those inhibitors (Additional file 1: Figure S7).

**Fig. 8:**
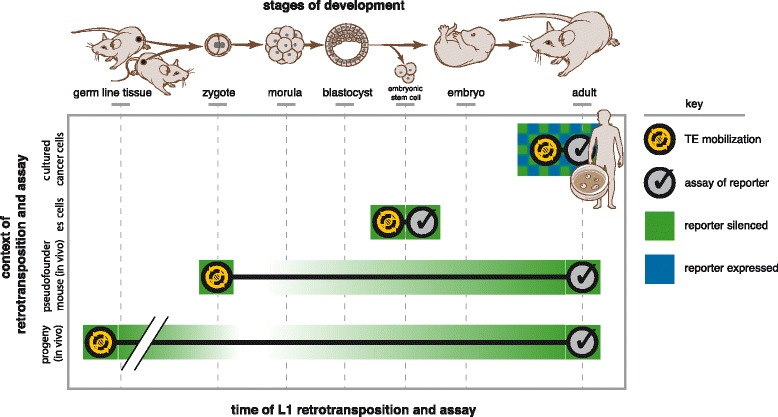
A model depicting differential expression and silencing of new L1 insertions, reflecting the cellular and developmental contexts of L1 integration. In this model, we propose that when L1 elements inserted during early development, in ES cells or before transmission through the germ line, such new insertions are densely methylated and silenced at the time of integration and when assayed subsequently. By contrast, if L1 mobilization occurred in adult somatic tissues such as cultured cancer cell lines, such L1 integrants undergo histone deacetylation and variegation, without cytosine methylation. This model is consistent with established expression differences of de novo methyltransferases Dnmt3a and Dnmt3b: robust levels were observed early in development and in ES cells, while low levels were described in differentiated somatic cells [63]. Additional factors could account for differential epigenetic regulation of newly inserted sequences in various developmental contexts. *Top*: various developmental time points as indicated. *Key: yellow circle*: time point when retrotransposition occurred; *black checkmark*: time when expression and methylation status of new TE insertions were assayed; *green*: silenced reporter; *white*: erasure of methylation and silencing; *green and blue checkerboard*: variegated reporter expression

We speculate that this variegated expression of reporter sequences retrotransposed by L1 in cultured cancer cells may reflect the timing of their integration, i.e., at later stages of somatic development (Fig. 8), when cellular *de novo* methyltransferases are expressed at low levels [60]. Retrotransposon silencing typically may be both incomplete and stochastic [59]. Thus the observed variegation in new reporter expression may be explained by this stochastic nature of L1 silencing. Although variegation typically is mitotically stable, the inheritance of a heterochromatic state may rapidly switch from a repressed chromatin state to an open state [61]. The variegated pattern of cells expressing high and low L1 reporter level observed here could be due to rapid changes in chromatin structure induced by L1 integration [62]. An alternative explanation for variegation of L1 reporter expression in cultured cancer cells could be related to genome-wide hypomethylation observed in most human cancers.

In contrast to the variegated, HDAC-mediated silencing of newly retrotransposed L1 integrants in cultured cancer cells, new L1 insertions in mouse ES cells were silenced by dense *de novo* CpG methylation (Figs. 5 and 8). Totipotent mouse embryonic stem (mES) cells can be viewed as a surrogate for the undifferentiated cells present in early embryos. Notably, abundant *de novo* methyltransferases are expressed in both ES cells and early postimplantation embryos [63]. In addition, we observed that new L1 integrants, present in the differentiated somatic tissues of adult pseudofounder mice, also were stably silenced by dense cytosine methylation (Figs. 6 and 8). This dense cytosine methylation may represent faithful maintenance of the initial epigenetic marks established very early during embryogenesis when the insertions occurred (Fig. 8). Dense cytosine methylation in mES cells may mimic epigenetic controls established in early development in vivo (Figs. 5 and 6). One plausible explanation is that *de novo* DNA methyltransferases, which are highly expressed in early embryogenesis and in ES cells [60], could target the newly inserted L1 cDNA sequences, which initially would be unmethylated at time of integration. In developing embryos, these enzymes normally re-establish DNA methyation after a wave of hypomethylation erases most methylcytosine marks. A recent study of extant L1 expression in human ES cells suggested that predominantly those elements localized in expressed genes were expressed, while others located outside of such genes were not [64]. However, this differential expression of L1 elements may reflect overlapping expression of flanking gene exons which would include intronic L1s, rather than the specific expression of intronic L1s per se. In addition, the activation of endogenous L1 expression, upon reprogramming somatic cells into induced pluripotent stem (iPS) cells, also implied that epigenetic derepression of silenced elements can occur [65]. By contrast, another group reported that silencing of TEs is stable during reprogramming of somatic cells to iPS cells [66].

We conclude that the distinct types of reporter expression and epigenetic regulation of new L1 insertions observed in mES cells or somatic tissues in vivo, in comparison with those in cultured somatic cells, could be related to the different cellular contexts or stages of differentiation in which L1 mobilization occurred initially (Fig. 8). We inferred that the observed epigenetic marks at *de novo* L1 integrants could reflect the developmental timing at which they integrated (Fig. 8), by defining the epigenetic marks established and then maintained at new integrants. Notably, we measured reporter expression and epigenetic silencing long after the time of retrotransposition per se, i.e., after many cell divisions. This limitation was due to current technical constraints, since initially the L1 donor elements had retrotransposed in individual cells that are experimentally inaccessible except by single cell cloning and sequencing, or upon embryonic development and tissue differentiation.

The resulting, distinct forms of silencing at newly integrated L1 reporters observed in various contexts may have important implications for the expression of the L1s themselves, for the regulation of other genes neighboring the new insertions, and for chromosomal architecture. We conclude that *de novo* L1 retrotransposition can contribute to significant variability in epigenetic marks established in cellular genomes.

In contrast to our observations about a lack of de novo methylation at newly inserted L1 seuqences, previous experiments have demonstrated that newly inserted foreign DNA can undergo de novo methylation in cultured somatic cells [67, 68]. Therefore, as a control, we used *PB* DNA transposons to mobilize the same reporter genes, both in cultured cancer cells and in mES cells (Fig. 7). Although our L1 or *PB* reporters are comparable to transgenic insertions of foreign DNA in various cellular contexts, there are several fundamental differences. For example, the target site preferences of L1 retrotransposition vs. *PB* transposition vs. random integrants of transgenesis are all distinct. While multiple copies of a transgene frequently can recombine at a single genomic locus [69], individual copies of L1 reporter genes typically integrate at interspersed genomic sites. We observed two key differences in silencing of *de novo* L1 reporter integrants vs. *PB* integrants. First, we observed minimal or no variegation of *PB* reporter expression; instead, their expression appeared to be mostly “all or none”. Second, the percentage of cells in which *PB* reporter integrants were silenced was much lower than that with variegated or silenced L1 integrants. These results also are consistent with a lack of *PB* integrant silencing observed in vivo [70, 71]. We speculate that these differences between L1 vs. *PB* reporter expression or silencing may reflect different genomic target site preferences of their mobilization. The differences also could be related to differences in the mechanisms of transposition by these elements. Thus, in comparison with the target sites of new L1 integrants, which are enriched slightly in intergenic genomic regions [72] or are distributed randomly [73], more than half of *PB* integrant sites are enriched inside expressed genes [74]. Alternatively, the PB-mobilized insertions would include PB inverted terminal repeats which could serve as insulators. However, a recent study showed that incorporation of bona fide insulator sequences flanking the transgenic PB reporter genes increased their expression [75]. This suggests that the PB sequences themselves may possess only minimal insulator activity.

In a recent study of epigenetic silencing of new L1 insertions in human embryonic carcinoma (EC) cells, histone deacetylation was identified as the silencing mechanism [31]. We note important similarities and differences between that study’s results and our data. We confirmed that histone deacetylation occurs at new L1 insertions, but only in cultured cancer cells and not in mES cells. We found that new L1 insertions were densely methylated in mES cells (Fig. 5), whereas new insertions in hEC cells were not silenced by cytosine methylation [31]. The discrepancy could be attributed to differences in the host cells’ species of origin; different epigenetic mechanisms operating in hEC vs. mES cells; distinct structures or sequences of the mobilized elements themselves; or various extents to which the cells had differentiated in vitro. Notably, ES and EC cells represent very different stages of differentiation [76], despite similar levels of expression of “stem cell-like” markers such as *OCT4*. ES cells are derived from the inner cell mass of developing embryos, while EC cells are derived from germ cell tumors. Compared with ES cells, the EC cell line PA-1 [31] represents a later stage of embryogenesis. Mouse EC cells have been shown to express full length RNA and ORF1p from pre-existing L1 elements [77]. However, a possible relationship between de-repressed chromatin in mES cells or hEC cells and the establishment of *de novo* epigenetic controls established at new L1 insertions is still unclear.

We also studied silencing of newly mobilized L1 insertions in vivo, both in differentiated tissues of pseudofounder mice and in their offspring. By contrast, the prior study did not include an analysis of silencing of new integrants in vivo [31]. In addition, the mobile genetic elements used as controls in the two studies to compare with L1 mobilization were very different. In the prior study, HIV-like retroviruses mobilized the transgenic reporter genes [31], whereas we used *PB*, a DNA transposon, to compare with L1 reporter silencing. These control vectors differ in their mechanisms of integration, genomic target sites, and the frequency of insertions generated per host genome. Each of these factors could play significant roles in shaping the downstream epigenetic silencing marks established at the new insertions.

We acknowledge potential limitations in our study. First, to investigate the state of expression and epigenetic regulation of newly inserted sequences that were mobilized by TEs, we artificially marked the L1 and *PB* donor elements using engineered, heterologous reporters including several different strong promoters and terminators. In comparison with native, unmarked elements, these reporter genes incorporated into donor TEs potentially could interfere with their mobilization. Moreover, upon integration they could trigger antisense transcripts [19] or otherwise artificially trigger or disrupt silencing by mimicking actively transcribed, protein-coding gene. A recent study noted that differences in promoters, reporter sequences or integration sites could influence reporter expression and thereby affect the study conclusions [78].

Second, we did not investigate L1 insertions that had newly integrated in germ line tissues. Extensive research has been conducted on the epigenetic control of pre-existing TEs in germ tissues during embryonic development. They appear to undergo a wave of demethylation followed by two distinct waves of *de novo* cytosine methylation [32, 79]. PIWI-interacting small RNAs (piRNAs), whose transcription is frequently initiated from TEs in germ tissues, mediate their regulation and silencing. Recent evidence also indicates that DNA methylation in hES cells is induced by PIWI/piRNA-mediated silencing. Small RNAs also may play important roles in guiding establishment of *de novo* methylation in somatic tissues [39]. We currently are studying roles of possible small RNAs, e.g., including those generated from antisense transcripts [19], in targeting *de novo* methylation.

Third, new insertions were not identified immediately after their integration in single cells. This approach has become technically possible recently, so we could identify and characterize new insertions in individual cells or very small subclonal populations within a few cell divisions of integration. Their minimal allelic fractions would require use of ultra-deep sequencing, making further analysis technically difficult but feasible for the first time.

Fourth, we did not perform chromatin immunoprecipitation (ChIP) experiments to assess enrichment of particular histone modfiications at the new L1 integrants, although we measured cytosine methylation in detail. However, in a recent paper describing L1 reporter silencing by histone deacetylation in hEC cells, confirmatory ChIP data [31] were strongly consistent with our HDAC drug inhibition studies (Fig. 4, Additional file 1: Figure S6).

The donor TE constructs used here did not include a means to terminate conditionally their capacity for ongoing retrotransposition. Future designs will incorporate this feature, to avoid the potential for ongoing genetic variability, e.g., after subcloning individual cells.

In some cases, rather than assay for reporter expression, we monitored retrotransposition by using PCR-based assays to identify genomic insertions of newly transposed reporter genes. As was the case with reporter expression assays used here, we did not select positively or negatively on cells harboring such inserted sequences to preferentially include expressed reporters or exclude silenced reporters.

And finally, although in vivo mouse models harboring control *PB* donor elements have been developed, whereby we could compare their silencing during development and in diverse tissues, such strains were unavailable to us. However, published studies have indicated that *PB* insertions typically are not silenced even when selection is not imposed [70, 80].

In summary, we showed here that the expression and silencing of newly integrated sequences mobilized by L1 retrotransposition appear to be associated with the cellular, developmental and genomic contexts of their integration. We hypothesize that the distinct epigenetic marks set up at new TE insertions that integrated in different cellular or developmental contexts may have various downstream consequences. For example, recent evidence suggests that most new somatic L1 insertions mobilized during human cancer development can mediate only minimal, if any, impacts on neighboring gene expression, unless they cause direct insertional mutagenesis of coding exons [11, 25]. By contrast, new insertions occurring early in development could more significantly disrupt the expression of neighboring genes, in part because their allelic fraction would be higher. In addition, the epigenetic silencing including cytosine methylation that is established at them would be expected to be more repressive and stable (Fig. 8). Thus we speculate that somatic TE that integrated early in development would exert stronger disruptive effects on neighboring genes, because more repressive epigenetic controls including methylcytosine marks would be established at them. These findings can be compared with those of Doerfler et al., who characterized de novo methylation established at foreign DNA introduced into mammalian cell genomes [67].

We propose that these findings may have important practical implications for evaluation and understanding of new TE insertions in various biological contexts. For example, they may facilitate a novel experimental assay of transposition timing, i.e., to identify when the elements mobilized in vivo. Thus we would expect to find dense cytosine methylation strongly repressing newly integrated, polymorphic L1 insertions that were mobilized early in development or passed through the germ line (Fig. 8). Such integrants might be present at a high allele fraction (e.g., 50%, in heterozygosity). By contrast, a somatic L1 polymorphism occurring later in development or differentiation would be expected to be mosaic, so it would be present at a much lower allelic fraction in one tissue and not another, such as in a tumor but not in matched normal tissues. This type of new insertion would be silenced more dynamically and reversibly by histone deacetylation. These epigenetic characteristics would suggest that its mobilization occurred in differentiated somatic cells.

## Conclusions

Analysis of newly inserted genomic sequences retrotransposed by L1 in various somatic cells and tissues revealed distinct patterns of expression and epigenetic regulation. In cancer cell lines, the newly retrotransposed integrants typically underwent rapid transcriptional gene silencing, but they lacked cytosine methylation, and their reporter expression was reversible and oscillated frequently. Silenced or variegated reporter expression was strongly and uniformly reactivated by treatment with inhibitors of histone deacetylation. By contrast, newly inserted sequences retrotransposed by L1 in mouse embryonic stem (ES) cells underwent rapid silencing by dense cytosine methylation. Similarly, *de novo* cytosine methylation at new integrants also was observed in several distinct somatic tissues of adult pseudofounder mice. We conclude that the host cellular and developmental contexts of retrotransposition are significant determinants of reporter expression and epigenetic silencing at newly integrated sequences mobilized by L1 retrotransposition. We have proposed a model whereby reporter expression of somatic TE integrants reflects the timing, molecular mechanism, epigenetic controls and the genomic, cellular and developmental contexts of their mobilization.

## Methods

### Plasmid constructs

To construct an L1 donor launched from an episomal plasmid, i.e., pDES46 (Fig. 1), where human L1 was marked by the *TEM1-*AI beta-lactamase/artificial intron (AI) reporter cassette inserted into its 3′ UTR, we first deleted the BamHI site in ORF2 of human L1.3 as present in pJM101/L1.3 (kindly provided by Dr. John V. Moran, Univ. Michigan) using site-directed mutagenesis. The L1.3/NeoR-AI promoter cassette was then moved into pBSII-KS using NotI and BamHI. We then introduced double-stranded oligonucleotides (containing Bst1107I -HSVTKpolyA-MluI) to replace the NeoR-AI and promoter fragments from pJM101/L1.3. BseRI sites were introduced to flank both *TEM1* (sequences obtained from the vector pBLAK-b which were then codon optimized) and beta-globin AI, using fusion PCR. Both of these constructs were cut out using BseRI and ligated together seamlessly. The resulting L1.3/TEM1-AI construct was then excised from pBSII-KS using NotI and BamHI and ligated into pCEP4 (Invitrogen), yielding pDES46.

As previously described, a conditionally activated mouse synthetic L1 donor, i.e., pJH435 (Fig. 5), was constructed by introducing a loxP-stop-loxP cassette between a strong composite promoter and the ORFeus L1 donor [54]. This construct consisted of a composite CMV immediate early enhancer and modified chicken beta-actin promoter; a floxed beta-geo/stop cassette comprised of a hybrid beta-galactosidase/neomycin phosphotransferase fusion gene and triple tandem copies of the SV40 late polyadenylation signal; L1 ORFeus ORF1 and ORF2 [56]; a GFP-based retrotransposition indicator cassette with its own promoter and polyadenylation signal; and beta-globin polyadenylation signal.

The constitutively active mouse synthetic L1 donor, i.e., pJL5, was prepared by cloning the *TEM1*-AI reporter cassette into the 3′ UTR of L1 ORFeus. Similar to pDES46, pJL5 also was cloned in the episomal plasmid pCEP4 backbone.

### Cultured cells

HCT116 (human colorectal carcinoma) cells, kindly provided by Drs. Ina Rhee, Christoph Lengauer and Bert Vogelstein (Johns Hopkins University), were cultured in McCoy’s 5A modified medium (Gibco, Life Technologies) supplemented with 10% heat inactivated fetal bovine serum (FBS) and 1% penicillin-streptomycin (P/S), at 37 °C and 5% CO_2_ in a humidified chamber. HeLa.JVM (a subclone of human cervical carcinoma) cells, provided by Dr. John V. Moran (University of Michigan), were cultured in Dulbecco’s Modified Eagle Medium (DMEM) with the same supplements as HCT116 cells. E14Tg2a.4 mouse embryonic stem (ES) cells, derived from 129P2 ES cells, were provided by Dr. Allan Bradley (Sanger Institute) and the BayGenomics resource. They were cultured without feeder cells in Glasgow’s Modified Eagle’s Medium (Sigma Aldrich) supplemented with 10% ES cell-qualified FBS, 1% non-essential amino acids, 1% L-glutamine, 1% sodium pyruvate, 0.1 mM 2-mercaptoethanol (2ME) and ESGRO (Millipore) at 1000U/mL in a 7% CO_2_ atmosphere.

Fugene 6 (Roche Applied Science, USA) was used to transfect the cultured cancer cell lines. Cells were transfected with pDES46 and were selected on Hygromycin (Invitrogen) at 0.3 mg/ml for 2 weeks. Resulting HygroR cells were cloned by limiting dilution into 96-well plates and screened for single colonies. Colonies were assayed for TEM1p beta-lactamase expression using the CCF2-AM assay [19]. Certain colonies were picked for further downstream analysis.

Mouse ES cells including Bruce4 cells (provided by Dr. Colin Stewart, NCI; [53]), and the resulting Truck_305 cells containing a mouse synthetic L1 ORFeus donor transgene (purchased from Ozgene [54]), were grown in high-glucose DMEM, supplemented with 15% ES-cell-qualified FBS, 1% L-glutamine, 1% non-essential amino acids, 0.1 mM 2ME, 50 U/ml of P/S and ESGRO in 10% CO_2_. For feeder cells, *Neo2*-expressing mouse embryonic fibroblasts (MEFs) were cultured in DMEM supplemented with 10% FBS and 1% P/S, in 5% CO_2_, arrested using Mitomycin C or gamma irradiation, and seeded in dishes. The mES cells were added 2–3 days after feeder cell seeding.

To activate L1 retrotransposition in these cells, an adenoviral vector encoding Cre recombinase was introduced to excise the *lacZ* LSL cassette. Briefly, one million Truck_305 cells were incubated for 30 min with the adenoviral Cre vector (Viral Technology Laboratory, NCI Frederick) in a 7% CO2 incubator, at various multiplicities of infection (MOIs) ranging from 10 to 200 per cell, and then plated into a well of a 6-well dish that was pre-seeded with mouse feeder (PMEF) cells. Cells were evaluated for cell death by light microscopy after 18 h incubation. Based on the extent of cell death and colony morphology, MOIs 25 and 50 were found to be optimal. Cell clones exposed to these MOIs were expanded. After at least 12 d in culture, with periodic changes of culture medium, cells were stained with crystal violet and X-gal, to visualize the colonies and assay for presence of the *lacZ* LSL cassette. ES cell clones that did not stain blue with X-gal, which indicated activated smL1 expression and potential retrotransposition, were propagated.

### Reporter assays

To quantify beta-lactamase activity and protein expression, cells were stained with CCF2-AM substrate (Life Technologies) [43] by replacing culture medium with 1 mL loading solution (2 μL of a 1 mM CCF2-AM solution, 16 μl of Solution B, 10 μl of 250 mM Probenicid (Sigma) and 972 μl Hanks’ Balanced Salt Solution, HBSS) per 9.6 cm2 well. Cells were incubated in the dark at room temperature for one hr with gentle shaking, washed with HBSS, and visualized using an Axiovert 200 M inverted fluorescence microscope (Zeiss) equipped with blue/aqua and beta-lactamase ratio filter sets (Chroma Technology Corp.) and either an ORCA-ER high resolution digital camera (Hamamatsu Photonics) using Openlab software (version 4.0.2, Improvision), or a Zeiss camera using AxioVision software. Flow cytometric analysis [47] was performed using a BD LSR II flow cytometer with a 405 nm violet laser, 440/40 nm (blue) and 530/30 nm (green) filters, and FACSDiva software (BD Biosciences). Ratios of blue to green intensities were collected as a linear parameter. Each flow cytometry session included positive and negative controls to normalize outputs.

### In vivo mouse models

Transgenic founder and pseudofounder mice were generated by pronuclear injection of linearized, marked smL1 donor cassette into fertilized eggs as described previously [55]. The unspliced L1 donor construct as well as spliced new genomic L1 insertions were identified by PCR amplification of the reporter gene, into and/or across the AI, respectively. Pseudofounder mice were defined by the presence of new spliced L1 insertions and the absence of the unspliced donor element. Their progeny were generated by backcrossing the founder and pseudofounder mice with wildtype mice. Certain *de novo* L1 integrants were transmitted in heterozygosity.

### Genomic DNA isolation and recovery of TE integrants

To extract genomic DNA, we added 600 μL lysis buffer with 420 μg/mL of proteinase K to cells growing in a 48 well plate. Lysis buffer consisted of 50 mM Tris-Cl, pH 8.0, 100 mM EDTA, 100 mM NaCl and 1% SDS. Next, 200 uL saturated NaCl was added, followed by an equal volume of isopropanol. After centrifugation, DNA pellets were washed with 70% ethanol, dried and resuspended in TE buffer.

Southern blotting was performed to identify lengths and amounts of genomic fragments harboring new TE insertions. Genomic DNA was electrophoresced, blotted, and probed for the *TEM1* reporter using standard methods.

For recovery of TE integrants in cultured cancer cells using inverse PCR (iPCR), genomic DNA isolated from clonal populations of cells was digested with a restriction enzyme (RE) such as XbaI, EcoRI or HindIII. Upon heat inactivation of the RE, digested products were diluted to 1 ng/μL in a total of 500 μL, and incubated with T4 DNA ligase overnight at 16 °C for intra-molecular ligation. After ethanol precipitation, DNA was resuspended and used in iPCR reactions using primers DES682 and DES209 which annealed to the 3′ end of the retrotransposed cassette. PCR reactions consisted of 40 cycles at 94 °C for 30 s, 55 °C for 30 s and 72 °C for 2 m 20 s. Each of the several bands observed by gel electropheresis after PCR were cloned into pCR2.1 using TOPO cloning kit (Invitrogen) and transformed into bacteria. Colony PCR using M13 forward and reverse primers was used to identify bacterial colonies containing cloned insert. PCR products were cleaned up and sequenced. To map *de novo* insertion sites, Sanger sequence reads were aligned against the reference human genome (hg19) using Blat.

To map new L1 integrants in mES cells using linear amplification-mediated (LAM-) PCR, we chose three ES cell clones (i.e., 1B6, 1C6 and 2B2) in which the spliced (i.e., retrotransposed) reporter gene had been identified by PCR assays. LAM-PCR reactions were set up with 50 ng gDNA from each ES cell clone, 2 nM dNTPs, 5 nM 5′-biotinylated primers DES3171 or DES3174, 1 uL of Advantage 2 enzyme in 1× buffer (Clontech), for 50 cycles (20 s at 95C, 45 s at 60C, and 90 s at 68C). Streptavidin-coated magnetic beads (200 mcg) were washed twice in 100 μL of binding buffer (1 M NaCl, 5 mM Tris, pH 7.5, 0.5 mM EDTA) using a magnetic separation stand, resuspended in 50 μL of 2× binding buffer, and mixed with the linear PCR reaction. The suspension was incubated for 60 min at RT under constant agitation, and then washed three times in 200 μL of wash buffer (10 mM NaCl, 5 mM Tris pH7.5, 0.5 mM EDTA and 0.01% Triton X-100). For second-strand synthesis, the matrix-bound DNA was resuspended in 20 μL of a reaction mixture containing 500 nM dNTPs, 100 ng/μL random hexamers, 5 U Klenow enzyme (New England Biolabs) and 1× NEB Buffer 2, and incubated at 37C for 60 min. After washing first with wash buffer and then twice in 1× reaction buffer, dsDNA was restricted using HaeIII or Sau3AI (NEB) at 37C for 2 hr, washed again in wash buffer followed by twice in 1× ligation buffer, and ligated with either HaeIII adapter (DES3177 and DES3178) or Sau3AI adapter (DES3177 and DES3179) using T4 DNA ligase at 16C overnight. To elute products, beads were resuspended in 5 μL of 0.1 N NaOH and incubated at RT for 10 min. The eluate was separated from the matrix using the magnetic stand, and was neutralized by adding 5 μL of Tris-Cl, pH 7.0.

To perform nested PCR, 1 μL of the eluate or from a 1/100 dilution from the first round of PCR was added as template in a second (nested) PCR reaction in 50 μL. Primers for the adapter (DES3181) and nested adapter (DES3182) were paired with DES3172 and nested primer DES3173 (HaeIII), or DES3175 and nested primer (Sau3AI), respectively, in the donor plasmid. Products were cloned into the Topo-TA pCR2.1 backbone (Invitrogen). To map integration junctions, Sanger sequencing was performed.

### Bisulfite sequencing

Bisulfite sequencing was performed using either the EZ DNA Methylation kit (Zymo Research) or the Qiagen Epitect kit, following the manufacturers’ instructions, respectively. Alternatively, we prepared fresh sodium hydroxide, sodium bisulfite and hydroquinone solutions to denature and treat DNA samples, which were then purified using Microcon-30 centrifugal spin columns (Amicon, Millipore) [81].

### Histone deacetylase (HDAC) inhibitors

The culture medium for cells growing at 50–70% confluence was replaced by medium containing one of HDAC inhibitors including TsA, scriptaid, apicidin, butyrate and nicotinamide (Sigma Chemical Co.) at the specified concentrations. After treatment for a specific time (12–24 h), the cells stained using the CCF2-AM assay and observed using a fluorescence microscope.

## Additional file

Additional file 1:
**Figure S1.** Schematic of beta-lactamase reporter assay. **Figure S2**. Schematics of newly integrated sequences retrotransposed by L1. **Figure S3**. Standardized blue and green cell populations as controls for flow cytometry analysis. **Figure S4.** Dense maintenance methylation of pre-existing L1 retrotransposons in cultured human colorectal cancer (HCT116) cells is reduced dramatically in *DNMT1* and *DNMT3b* methyltransferase double knockout cells. **Figure S5.** Lack of cytosine methylation at silenced, de novo L1 reporter insertions in cultured HeLa cells. **Figure S6.** Variable L1 reporter expression in cultured cancer cells is associated with changes in histone acetylation. **Table T1.** Oligonucleotides used in this study. **Table T2.** *De novo* L1 integrant features. **Table T3.** Expression status of predicted SAGE tags from consensus human L1 template sequence in sense and antisense orientation. (PDF 18544 kb)

## Abbreviations

2ME: 2-mercaptoethanol
AI: Artificial intron
ChIP: Chromatin immunoprecipitation
DKO: Double knockout
EC: Embryonic carcinoma
ERV: Endogenous retrovirus
ES: Embryonic stem
FBS: Fetal bovine serum
FRET: Fluorescence resonance energy transfer
GFP: Green fluorescent protein
GFPuv: Green fluorescent protein derivative tuned to UV light excitation
HDAC: Histone deacetylase
hES: Human embryonic stem
iPCR: Inverse polymerase chain reaction
LAM-PCR: Linear amplication-mediated polymerase chain reaction
MEFs: Mouse embryonic fibroblasts
mES: Mouse embryonic stem
NF: Not found
ORF: Open reading frame
P/S: Penicillin/streptomycin
PB: PiggyBac
piRNA: PIWI-interacting small RNA
RE: Restriction endonuclease (enzyme)
RIFT: Retrotransposon initiated fusion transcript
RT: Room temperature
TE: Transposable element
TGS: Transcriptional gene silencing
TsA: Trichostatin A
TSD: Target site duplication
UTR: Untranslated region
WT: Wildtype
