# Supplementary material for: Dynamic silencing of somatic L1 retrotransposon insertions reflects the developmental and cellular contexts of their genomic integration
Source: Mob DNA. 2017 May 10;8:8. doi: 10.1186/s13100-017-0091-2 (PMC5424313; doi:10.1186/s13100-017-0091-2)
Supplement: Additional file 1: — Figure S1. Schematic of beta-lactamase reporter assay. Figure S2. Schematics of newly integrated sequences retrotransposed by L1. Figure S3. Standardized blue and green cell populations as controls for flow cytometry analysis. Figure S4. Dense maintenance methylation of pre-existing L1 retrotransposons in cultured human colorectal cancer (HCT116) cells is reduced dramatically in DNMT1 and DNMT3b methyltransferase double knockout cells. Figure S5. Lack of cytosine methylation at silenced, de novo L1 reporter insertions in cultured HeLa cells. Figure S6. Variable L1 reporter expression in cultured cancer cells is associated with changes in histone acetylation. Table T1. Oligonucleotides used in this study. Table T2. De novo L1 integrant features. Table T3. Expression status of predicted SAGE tags from consensus human L1 template sequence in sense and antisense orientation. (PDF 18544 kb) [file 13100_2017_91_MOESM1_ESM.pdf]

SUPPLEMENTARY INFORMATION – KANNAN ET AL.

SUPPLEMENTARY FIGURES

Supp. Fig. S1. **Schematic of beta-lactamase reporter assay.** (A – C) As described by Zlokarnik et al. [43], a fluorescent substrate called CCF2-AM is taken up inside cells where it can be trapped by cytoplasmic esterases. Cleavage of the intracellular fluorescent substrate CCF2 is mediated by the reporter protein, beta-lactamase, which is encoded by the *TEM1* reporter gene. This cleavage of CCF2 changes its fluorescence emission from green to blue. The amount of blue emission correlates with beta-lactamase levels. From [43]. Reprinted with permission from AAAS. (D) Schematics of (*top*) full-length L1 donor with *TEM1*-AI reporter at 3' end, and (*bottom*) a hypothetical, newly inserted sequences mobilized by L1 retrotransposition in genomic DNA, including spliced TEM1 reporter and target site duplications (TSD, red filled circles). At bottom, a truncated integrant lacking full-length TEM1 reporter, so functional beta-lactamase is not expressed. Variable expression of beta-lactamase translated from the integrated TEM1 gene (from low to high) in individual cells results in different CCF2 fluorescent emissions ranging from green to blue.

Supp. Fig. S2**. Schematics of newly integrated sequences retrotransposed by L1. (A-C)** Each insertion shows characteristics of bona fide L1 retrotransposition, including poly(A) tails at the 3' end, (A, C) target site duplications (TSDs) at the 5' and 3' ends, and in (B), an inversion. We recovered an intact reporter gene after splicing and integration (A), while the others had truncations at the 5' end (B, C). Chromosomal coordinates of the integration sites are shown.

Supp. Fig. S3. **Standardized blue and green cell populations as controls for flow cytometry analysis.** Green cells (untransfected cells stained with CCF2-AM) and blue cells (variegating cells treated with 100nM Trichostatin A for 24 hrs; cf. Fig. 3 and following) were used to set the gates for analysis of the variegated cell populations. We used a Becton-

Dickenson LSR II flow cytometer fitted with a 405nm violet laser and 440/40nm (blue) and 530/30nm (green) filters. Scatter plots of cells' fluorescence emissions are shown; blue/green ratios were calculated for each cell's fluorescence emissions.

Supp. Fig. S4. **Dense maintenance methylation of pre-existing L1 retrotransposons in cultured human colorectal cancer (HCT116) cells is reduced dramatically in *DNMT1* and *DNMT3b* methyltransferase double knockout cells.** *Top*: Schematic of pre-existing, full-length L1 integrants interspersed genome-wide, including open reading frame-1 (ORF1) and ORF2 (*yellow arrows*). *Black square, number 1*: position within the 5' L1 untranslated region (UTR; *green*), analyzed by bisulfite sequencing. Each circle represents a CpG dinucleotide. *Filled circles*: methylcytosine; *open circles*: unmethylated cytosines; *red fill*: not determined. Each row represents a clone (allele) that was sequenced. Dense cytosine methylation was observed in individual alleles of the L1 5' UTR in (*top*) HCT116 parental cells but not in (*bottom*) double knockout (DKO) cells lacking both *DNMT1* and *DNMT3b* methyltransferases (*brackets labeled at right*). *Blue bars*: position of PCR amplicon to quantify methylation at CpG dinucleotides in L1 5' UTR genome-wide. Percentages of all CpGs in the 5'UTR amplicon that were methylated were calculated as 63.7% in the HCT116 parental cells and 6.5% in DKO cells, respectively. Percentage methylation at four critical CpGs at L1 5' UTR poisitons +52, +58, +61 and +70 (*blue bar*) were 72.4% in HCT116, and 17.7% in DKO cells respectively.

Supp. Fig. S5. **Lack of cytosine methylation at silenced, de novo L1 reporter insertions in cultured HeLa cells.** Cytosine methylation was assessed by bisulfite sequencing at newly mobilized L1 integrants in cloned HeLa cells, both (A) in the body of the *TEM-1* beta-lactamase reporter gene (*blue arrows*; bisulfite primers DES709 x DES519), and (B) in the SV40 promoter that drives the reporter (bisulfite primers DES711 x DES519). Each circle represents a CpG

dinucleotide. A solid circle represents a methylated dinucleotide, while an open circle represents an unmethylated one. Each row represents a clone (allele) that was sequenced.

Supp. Fig. S6. **Variable L1 reporter expression in cultured cancer cells is associated with changes in histone acetylation.** HeLa cells harboring de novo L1 reporter integrants were assayed for reporter beta-lactamase expression by incubating them with the fluorescent substrate, CCF2-AM. *Left*: before and *right*: after incubation for 24 h with various histone deacetylase inhibitors including: (A) 1 uM scriptaid and (B) 5 mM nicotinamide respectively.

Supp. Fig. S7. **De-repression and re-repression of L1 reporter gene upon treatment and removal of HDAC inhibitor.** Variegated HeLa cells (A) were treated with 100 nM Trichostatin A for 24 hrs, after which they were stained with CCF2-AM and visualized (B), when the population was entirely blue. Subsequently, after washing out TsA, the cells were visualized after an additional 8 hrs (C), 26 hrs (D) and 56 hrs (E) of culture. Upon removal of the histone deacetylase inhibitor, L1 reporter variegation gradually was re-established in the cell population (compare panels A, D and E).

SUPPLEMENTAL TABLES

Supp. Table T1. **Oligonucleotides used in this study.**

Supp. Table T2. ***De novo* L1 integrant features.** Human L1.3, marked by TEM1 reporter gene in pDES46 (Fig. 1), was mobilized in HeLa cells. Several cellular subclones were isolated by limiting dilution. Individual integrants were recovered using the L1 insertion dimorphism display method [44] which involves inverse PCR. Listed here are the chromosomal band and coordinates (hg17) of each insertion, the restriction enzyme used for their recovery, and

genomic features at the site of integration. Not listed are the sites of additional new L1 integrants (cf. Supp. Fig. S2).

Supp. Table T3. **Expression status of predicted SAGE tags from consensus human L1 template sequence in sense and antisense orientation.** SAGE tags mapping to L1.3, identified in HCT116 parental (WT) and double knockout (DKO cell) libraries. *Column headers*: *Tags*, predicted L1 sequence in tag; *WT*, normalized tag count (tags per million) in HCT116 library; *DKO,* normalized tag count (tags per million) in library DKO2L prepared from DKO cells; *Ratio DKO/WT*: ratio of tag counts expressed in DKO vs. WT; *AvgLong,* cumulative average count of the tags in all long-SAGE libraries on NCBI portal; *nt. position*: nucleotide position of the tags mapped to the L1.3 sequence in sense orientation, NF: tag not found. To compare the expression of tags in both libraries (DKO/WT ratios) in cases where tags were detected in one library but not the other, we substituted a normalized “half tag” expression value (normalized per million), i.e. 4.3 or 4.9 tags per million.

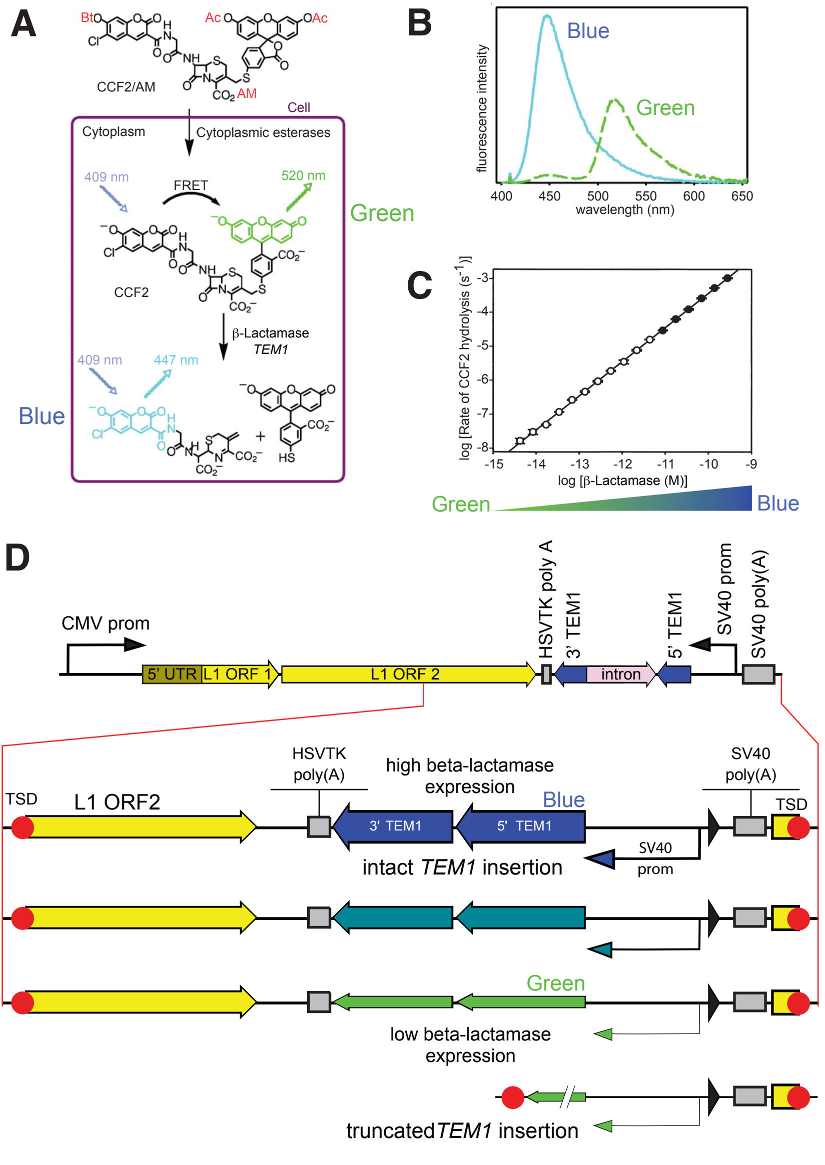

Kannan et al., Supp. Fig. 1

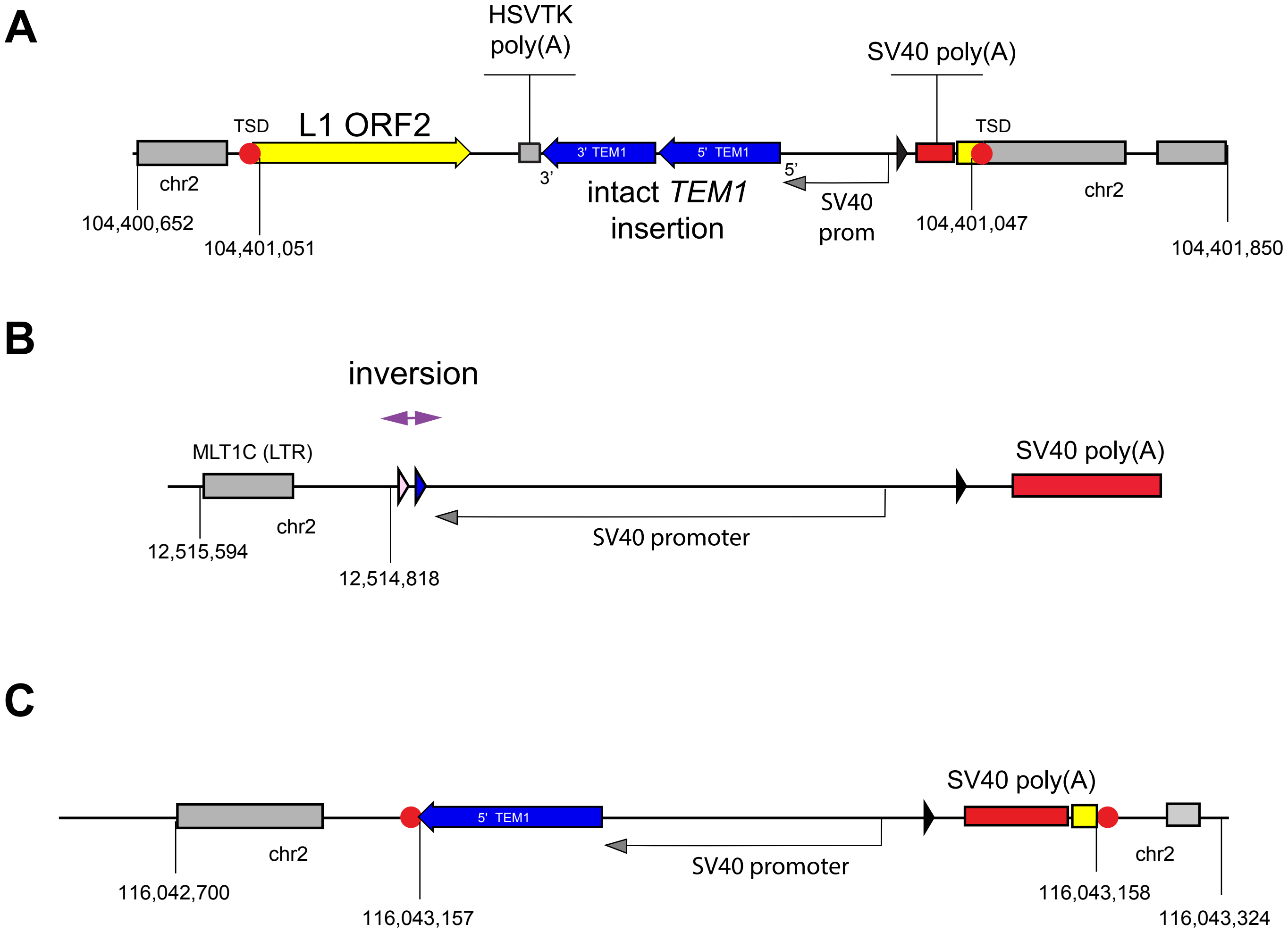

Kannan et al., Supp. Fig. S2

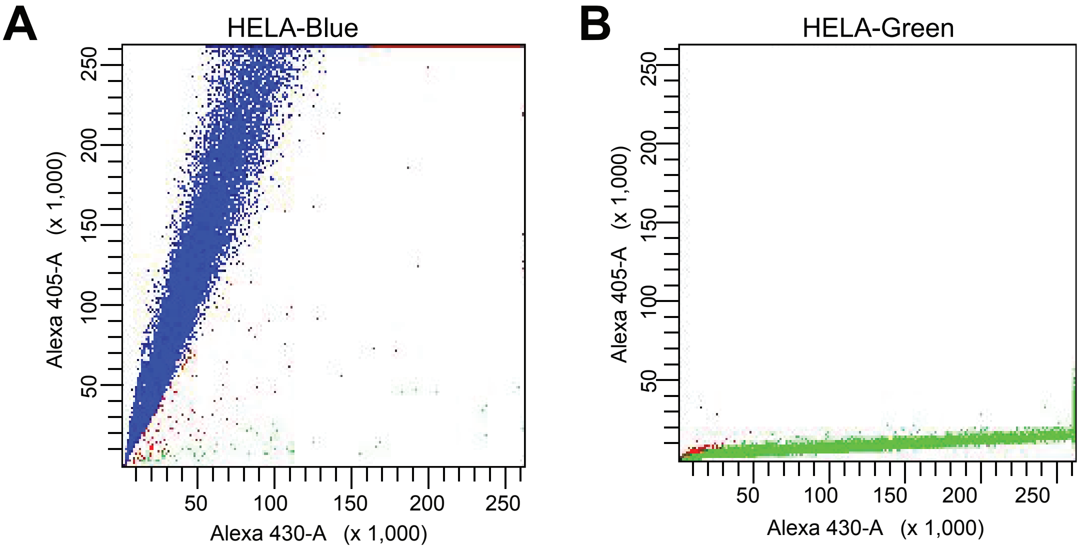
A
HELA-Blue
Alexa 405-A (x 1,000)
Alexa 430-A (x 1,000)
50
100
150
200
250
B
HELA-Green

Supp. Fig. S3

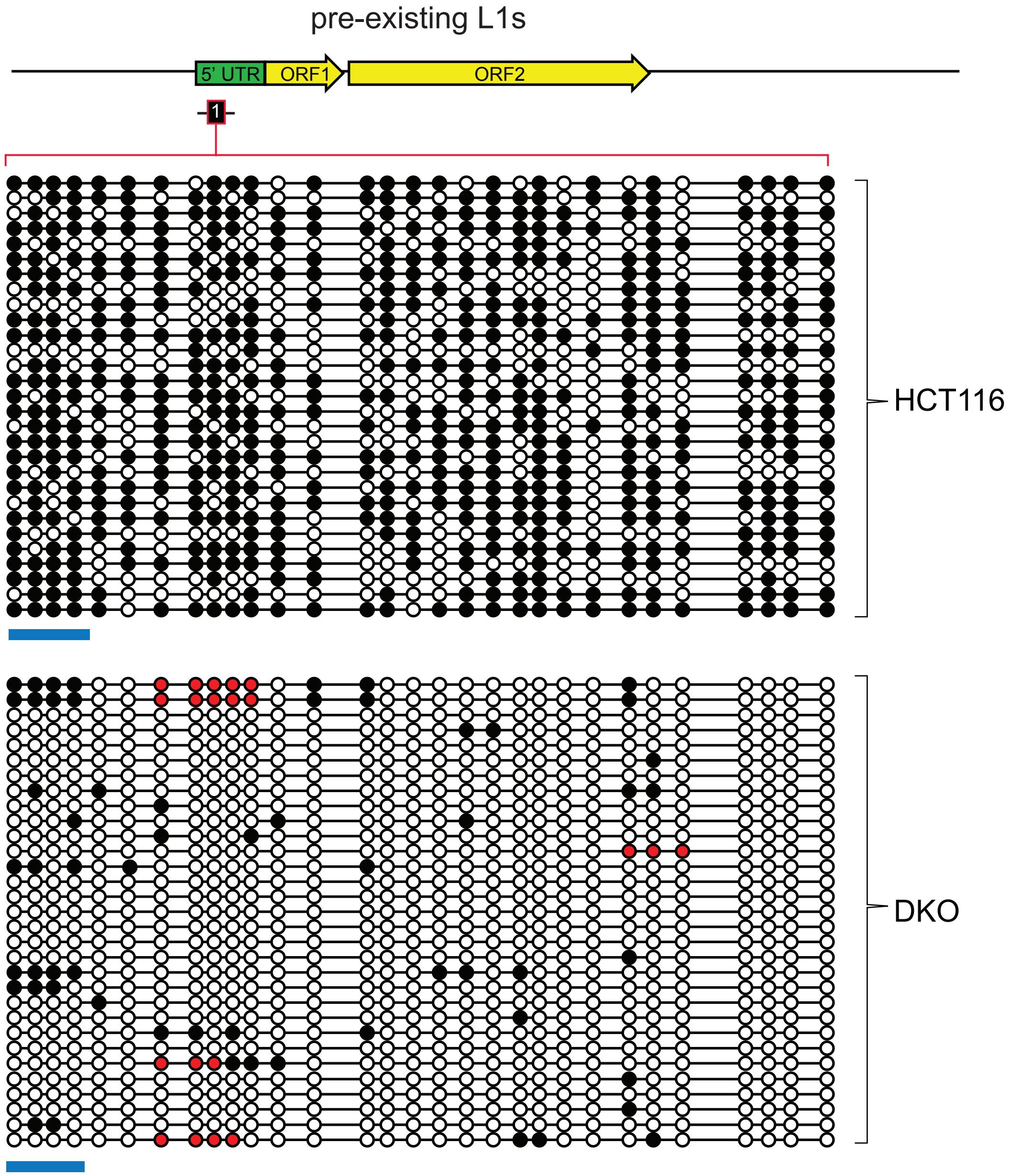

Supp. Fig. S4

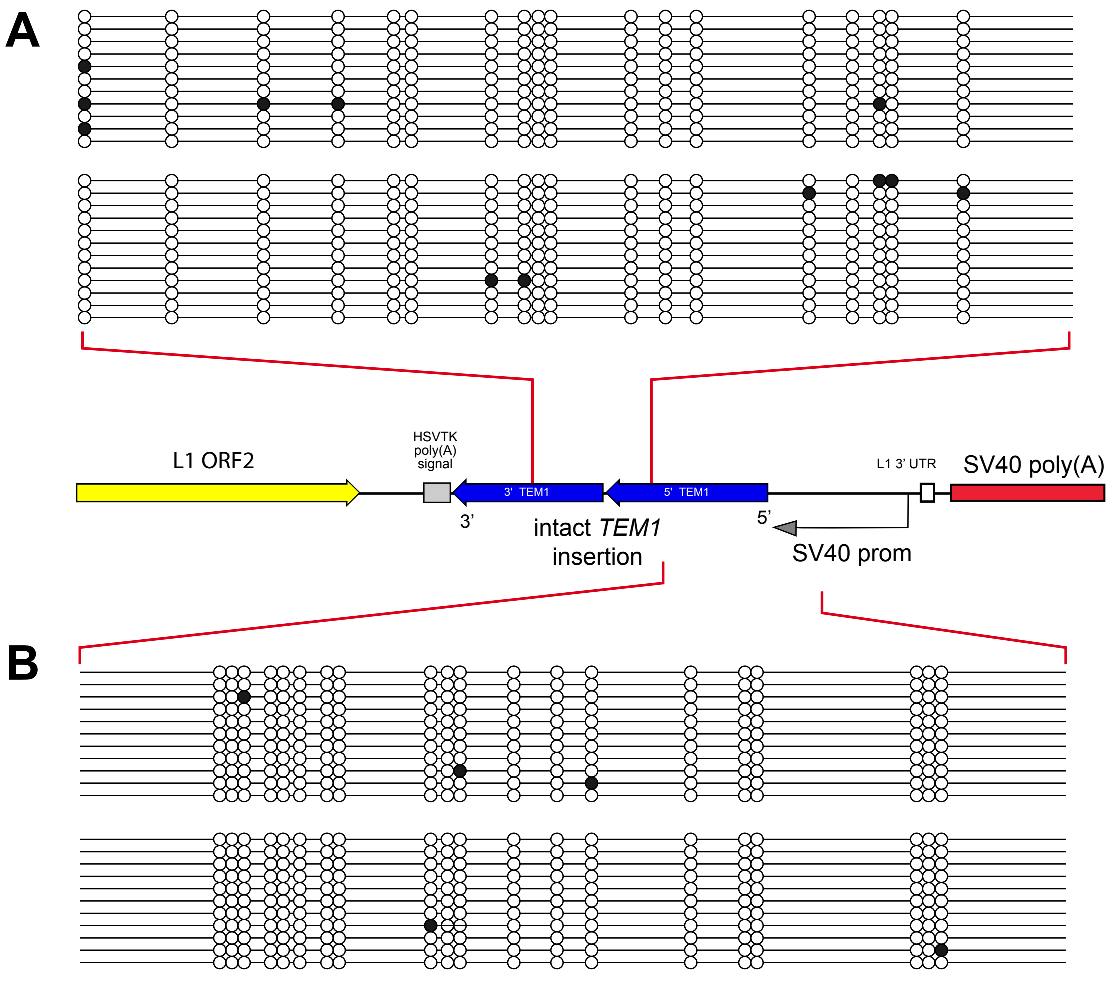

Kannan et al., Supp. Fig. S5

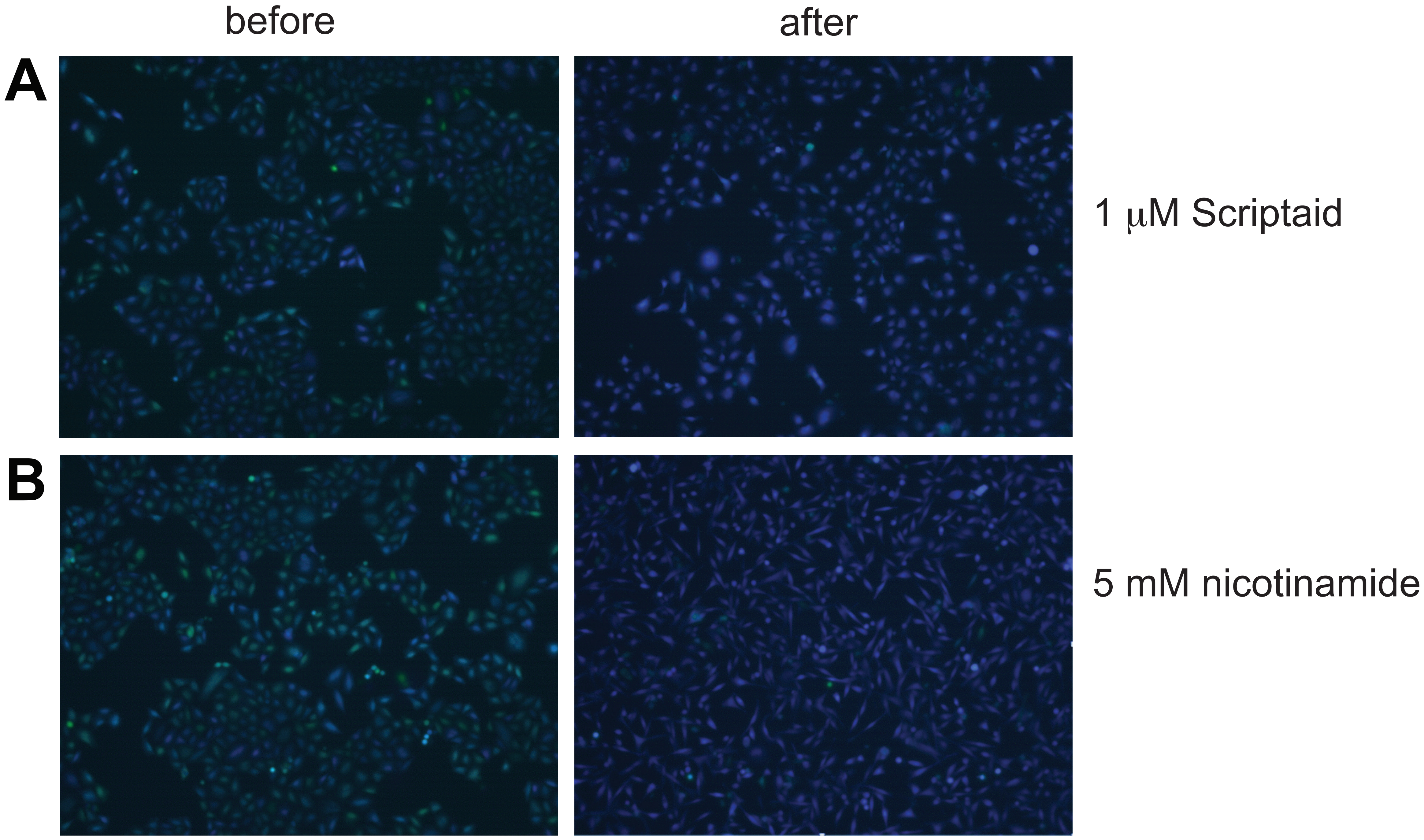

Kannan et al., Supp. Fig. S6

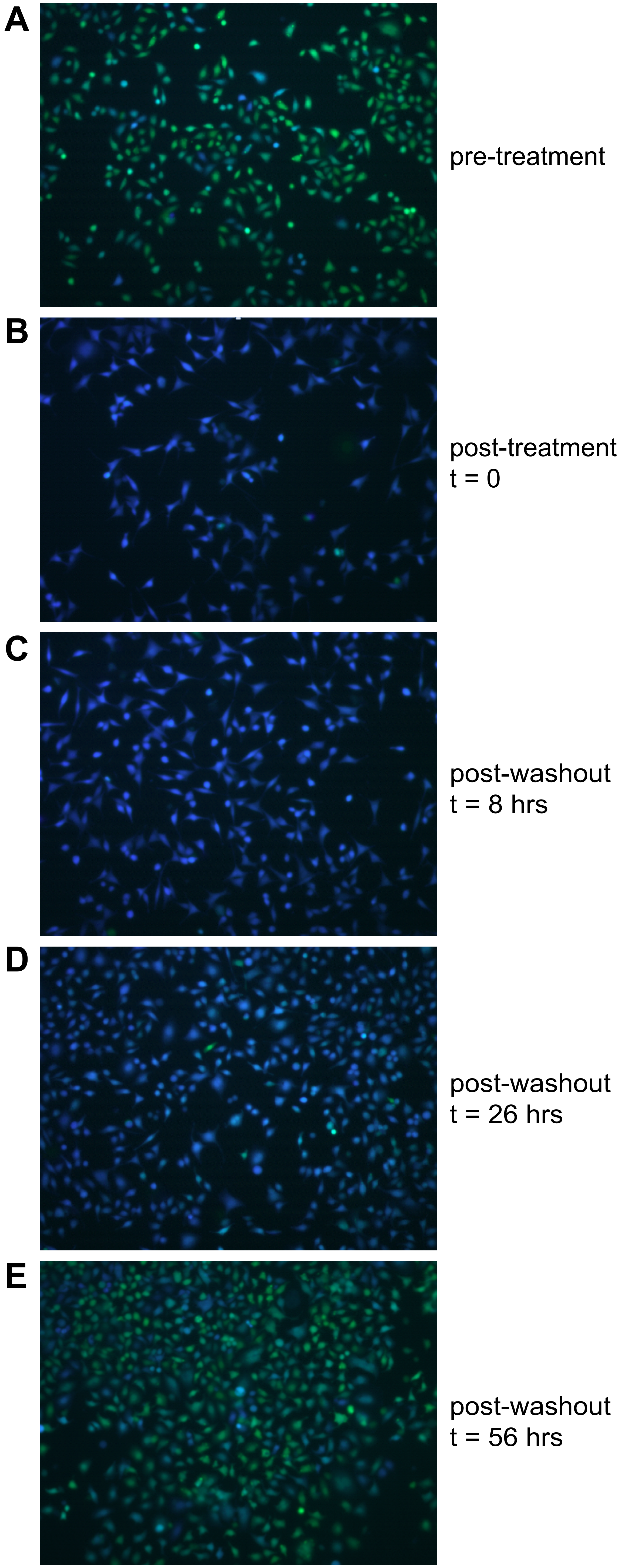
A
pre-treatment
B
post-treatment
t = 0
C
post-washout
t = 8 hrs
D
post-washout
t = 26 hrs
E
post-washout
t = 56 hrs

Supp. Fig. S7

Supp. Table T1	Oligonucleotide primers used in this study.	
DES209	5’-AGCTATTCCAGAAGTAGTG-3’	sense, within SV40 promoter in pCEP4, pDES46 backbone
DES459	5’-CCCCCAAAAATAAAACCTACAAAAAC-3’	Antisense L1 5’ UTR bisulfite sequencing primer, anneals at 556-531 of L1.3
DES460	5’-GTYGAATAGGAATAGTTTYGGTTTATAGTTTTTAG-3’	Sense L1 5’ UTR bisulfite sequencing primer, anneals at 18-45 of L1.3
DES512	5’-AAGAAAGGtTttTGATtTtTAGAAGtTGGGTAt-3’	HCT116 cell line 7H2, bisulfite sense, in genomic flanking sequence ~ 80 bp upstream of L1 5' UTR junction including CMV prom
DES515	5’-GGtAAGtAGGtATyGttATGGGTt-3’	bisulfite sense, within *Neo* gene
DES519	5’-CATTCTCCaCCCCATaaCTaAC-3’	bisulfite antisense, ~150 bp downstream of *Neo* in SV40 early promoter
DES524	5’-TCCTATCATCTCACCTTACTCC-3’	bisulfite antisense within *Neo*, at nt 6542-6521 of pDES89/spliced L1
DES530	5’-CAAATAAAACAATACCTCRCCCTA-3’	HCT116 cell line 7H2, bisulfite antisense L1 5’ UTR
DES657	5’-AATGCTTAATCAGTGAGGCACCTAT-3’	beta lactamase sense
DES658	5’-CAGAAACGCTGGTGAAAGTAAAA-3’	beta lactamase antisense
DES682	5’-GGGACTTTCCACACCCTAA-3’	antisense, within SV40prom promoter in pCEP4, pDES46 backbone
DES709	5’-yGtAGAAGTGGTttTGtAAtTTTATt-3’	bisulfite 3’ *TEM1* in pDES46 – sense strand
DES711	5’-GATGtTTTTtTGTGAtTGGTGAGTAtTtAA-3’	bisulfite 5’ *TEM1* in pDES46 sense strand
DES713	5’-CAaTaCTaCCATAACCATaAaTaATAACACT-3’	antisense *TEM1* primer in 5' end
DES2016	GAAGTATTTGAAGAAGTGTAGTATTAGTTTG	Bisulf. mod. sense primer located in smL1 ORF2

DES2018	CTTCTAAAAAAAAACATAATATCCACACTTC	Bisulfite modified AS primer located downstream of smL1 ORF2
DES2219	5'-GTTGGGGTTTTTGTTTAGGG-3'	sense, bisulfite seq in smL1 EGFP reporter
DES2221	5'-TAAATTAACAACATACCTTAC AAAAAAAAAAAAAACAC-3'	antisense, bisulfite seq in smL1 RSV promoter
DES3171	5'-bio-TCCTCGCCCTTGCTCACCAT3'	LAM-PCR linear amplification for integrant recovery in ES cells
DES3172	5'-ATGTGGTGAATGGTCAAATGGCG-3'	nested PCR for integrant recovery in ES cells
DES3173	5'-ACCCGTCTGTTGCCTTCCTAA-3'	nested PCR for integrant recovery in ES cells
DES3174	5'-bio-TTTCCCTCTGCCAAAAATTATGGG-3'	LAM-PCR linear amplification for integrant recovery in ES cells
DES3175	5'-ATGAAGCCCCTTGAGCATCTG-3'	nested PCR for integrant recovery in ES cells
DES3176	5'-CTTGAGCATCTGACTTCTGGC-3'	nested PCR for integrant recovery in ES cells
DES3177	5'-AGGTAACGAGTCAGACCACCGACT CGTGGAGGTTAGACTG-3'	HaeIII adapter and Sau3AI adapter in LAM-PCR for integrant recovery in ES cells
DES3178	5'-CAGTCTAACCTCCAC-3'	HaeIII adapter in LAM-PCR
DES3179	5'-GATCCAGTCTAACCTCCAC-3'	LAM-PCR adapter for integrant recovery in ES cells
DES3181	5'- AGGTAACGAGTCAGACCACC -3'	Sau3AI adapter in LAM-PCR for integrant

		recovery in ES cells
DES3182	5’ -GACTCGTGGAGGTTAGACTG-3’	nested PCR for integrant recovery in ES cells
DES3298	5’-GTAGTTGTTGAATATTTTATTT TTTGGTTAGAATG-3’	truncated GFPuv / RFP promoter insertion in 1B6-A08 ES cell clone
DES3299	5’-CCCAACTTTCTTATACAAAAT AATCCCC-3’	truncated GFPuv / RFP promoter insertion in 1B6-A08 ES cell clone
DES3301	5’-CACCCTCRTAACCACCTTCAA-3’	within GFPuv (not crossing splice site) in 1B6-A07 ES cell clone
DES3314	5’-GTGGTTGTTGTAGTTGTATTTT AGTTTGTG-3’	within GFPuv (not crossing splice site) in 1B6-A07 ES cell clone
DES3321	5’-GTTGGGGTTTTTGTTTAGGG-3’	crosses GFPuv-AI splice junction in 1B06/B02, 1C6 and 2D4 ES cell clone
DES3322	5’-AACATCCTAAAACACAAACTA AAATACAAC-3’	crosses GFPuv-AI splice junction in 1B06/B02, 1C6 and 2D4 ES cell clone

Chromosome locus (strand orientation)	Coordinates (hg17)	Enzyme used for recovery	Genomic context	Other
1q31.2 (-)	189,998,816	XbaI	LINE L1ME2	
2p24.3 (-)	12,515,594	XbaI	Between LTR1B and MLT1C	Supp. Fig. S1B
2q12.1 (+)	104,401,046	BclI	Between AluSc and LTR33	Supp. Fig. S1A
2q14.1 (+)	116,043,157	EcoRI	5th intron of DPP10 (dipeptidyl peptidase)	Supp. Fig. S1C
3q13.31 (+)	118,798,663	XbaI	Region 20bp upstream of GA repeat	
6q22.31 (+)	121,035,080	XbaI	Region 55bp upstream of AT repeat	
10q21.1		Sau3AI	Near L1MCa transposon	
t(11;13) putative translocation 11p15.4 (+); 13q21.32 (-)	11: 5,231,999; 13: 65,685,917	XbaI	chr11: intron of gamma globin gene HBG2<br>chr13: 150bp downstream of L1PB4	
17q24.1 (+)	60,690,241	EcoRI	SINE element MIRb	

Supp. Table T2.

Supp. Table S3 – SAGE tags mapping to L1.3, identified in HCT116 parental (WT) and double knockout (DKO cell) libraries.

Sense orientation

Tag	WT	DKO	DKO/WT	AvgLong	L1 Position
GAAAGGAACAACCGGTA	NF	NF	---	2.43	1879
CCAAAATGTAAAGACCA	4.3	9.8	2.2	1.62	1916
GAAACTGAACAACCTGC	4.3	49.08	11.2	29.7	2739
GAGGAACTGGTACCATT	NF	NF	---	0.27	3445
ATCAAGTGGGCTTCATC	NF	NF	---	1.62	3674
ATTATCTCAATAGATGC	NF	NF	---	0.54	3775
CTAAAAACTCTCAATAA	NF	NF	---	0.54	3829
ATTGTATATCTAGAAAA	NF	NF	---	0.27	4102
GGTGAACTCCCATTCGT	NF	NF	---	0	4252
GGTAGGAAGAATCAATA	NF	NF	---	0.54	4407
GTACTGGTACCAAAACA	NF	NF	---	5.6	4653
TCCAAAACACCAAAAGC	NF	NF	---	1.35	4975
GGAGAAAATTTTCGCAA	NF	NF	---	0	5097
AACAGACACTTCTCAAA	NF	NF	---	1.35	5218
AAGAAATGCTCATCATC	NF	NF	---	0.27	5266
CTGCTATAAAGACACAT	NF	NF	---	0	5550
CACACGTATGTTTATTG	NF	NF	---	2.43	5568
GAATACTATGCAGCCAT	NF	NF	---	0.54	5677
GATGAAATTGGAAACCA	NF	NF	---	0.54	5730
GACACAGGAAGGGGAAT	4.3	9.8	2.2	1.08	5834
TATACATATGTAACTAA	NF	NF	NA	5.13	5964
TACCCTAAAACTTAGAG	26.2	78.5	2.9	76.41	6000

Antisense orientation

Tag	WT	DKO	DKO/WT	AvgLong	L1 Position
TGCACATTGTGCAGGTT	NF	NF	---	0.81	65
TGCCATGCTGGTGCGCT	NF	NF	---	0	101
CTGGTGCGCTGCACCCA	NF	NF	---	11.8	108
TGATCTCATTGTTCAAT	NF	NF	---	0.54	231
TCCCTACAAAGGATATG	NF	NF	---	0.27	335
GTGTATATGTGCCACAT	4.3	9.8	2.2	4.3	388
TGTCTTTATAGCAGCAT	NF	NF	---	0	497
ATTTATACTCATTTGGG	NF	NF	---	0	515
TGTTTTTTGGCTGCATA	NF	NF	---	0.81	799
TCCTTCGCCCACTTTTT	NF	NF	---	2.7	847
TTGTAGGTTGCCTGTTC	NF	NF	---	0	968
AAGTCCTTGCCCACGCC	NF	NF	---	0	1090
CTGTTTTGGTTACTGTA	NF	NF	---	3.2	1412
GAATGTTCTTCAGCATG	NF	NF	---	0	1660
GAATGTTCTTCCATTTG	NF	NF	---	0.27	1677
ATTTGGCTCTCTGTTTG	NF	NF	---	0.81	1826
TCGTCTGCAAACAGGGA	NF	NF	---	0.54	1976
AAGGGTTGTTGAATTTT	NF	NF	---	0	2249
TGGTTTTTGTCTTTGGC	NF	NF	---	0.27	2303
GTGGATAAGCTTTTTGA	NF	NF	---	2.7	2404
TACCTCTGGTAGAATTC	NF	NF	---	0	2633

TAGTTGAGCGGCTTTGA	NF	NF	---	0.27	3339
ATTTTGCAGCGGCTGGT	NF	NF	---	0.27	4162
TTTAGCGCTTCCTTCAG	8.7	4.9	0.5	2.16	4199
